# Supplementary material for: Automated NMR resonance assignments and structure determination using a minimal set of 4D spectra
Source: Nat Commun. 2018 Jan 26;9:384. doi: 10.1038/s41467-017-02592-z (PMC5786013; doi:10.1038/s41467-017-02592-z)
Supplement: Supplementary file 1 — Supplementary Information [file 41467_2017_2592_MOESM1_ESM.pdf]

Supplementary Figure 1

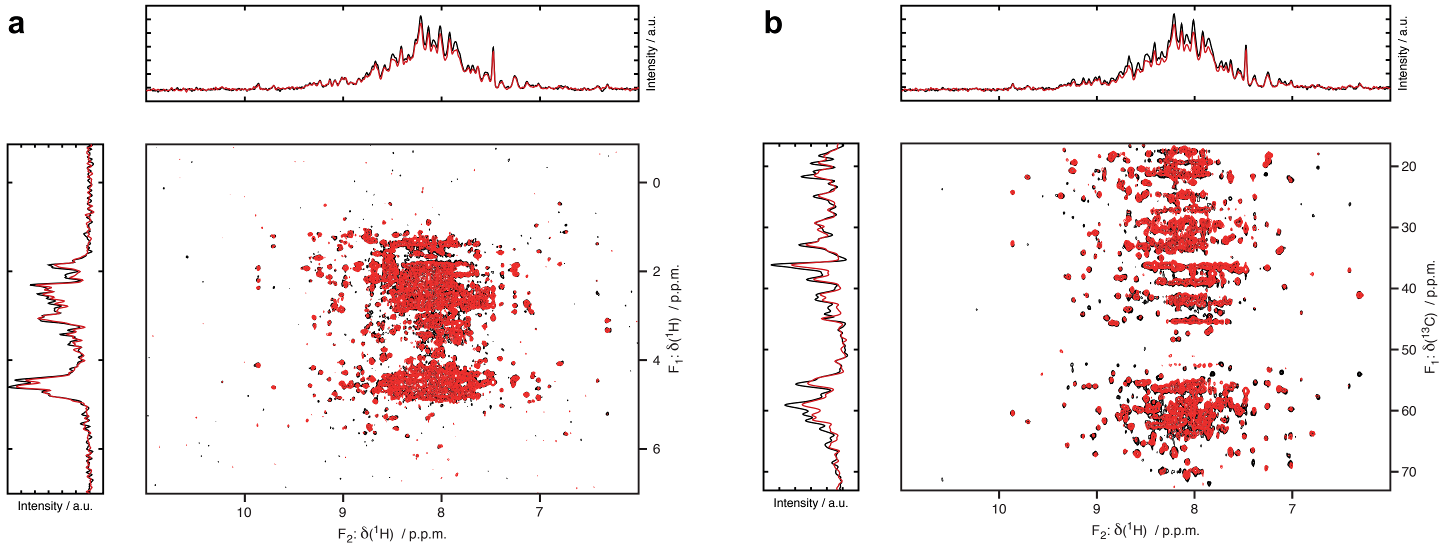

Supplementary Figure 1. Comparison of the signal strength for the 3D and 4D TOCSY pulse sequences. Sample is uniformly $^{15}N/^{13}C$-labeled nEIt protein of 27.3 kDa. 2D Haliph-HN (**a**) and 2D Caliph-HN (**b**) projection spectra measured using standard 3D HCCCONH TOCSY experiments (black) and 4D HC(CC-TOCSY(CO))NH used in our study (red). 1D ^{1}H and ^{13}C projections show ~5-10% lower sensitivity in case of the 4D experiment which are due to the different and longer CC-TOCSY mixing sequence used in the 3D spectra (DIPSI-2) and 4D experiment (FLOPSY-16), respectively.

Supplementary Figure 2

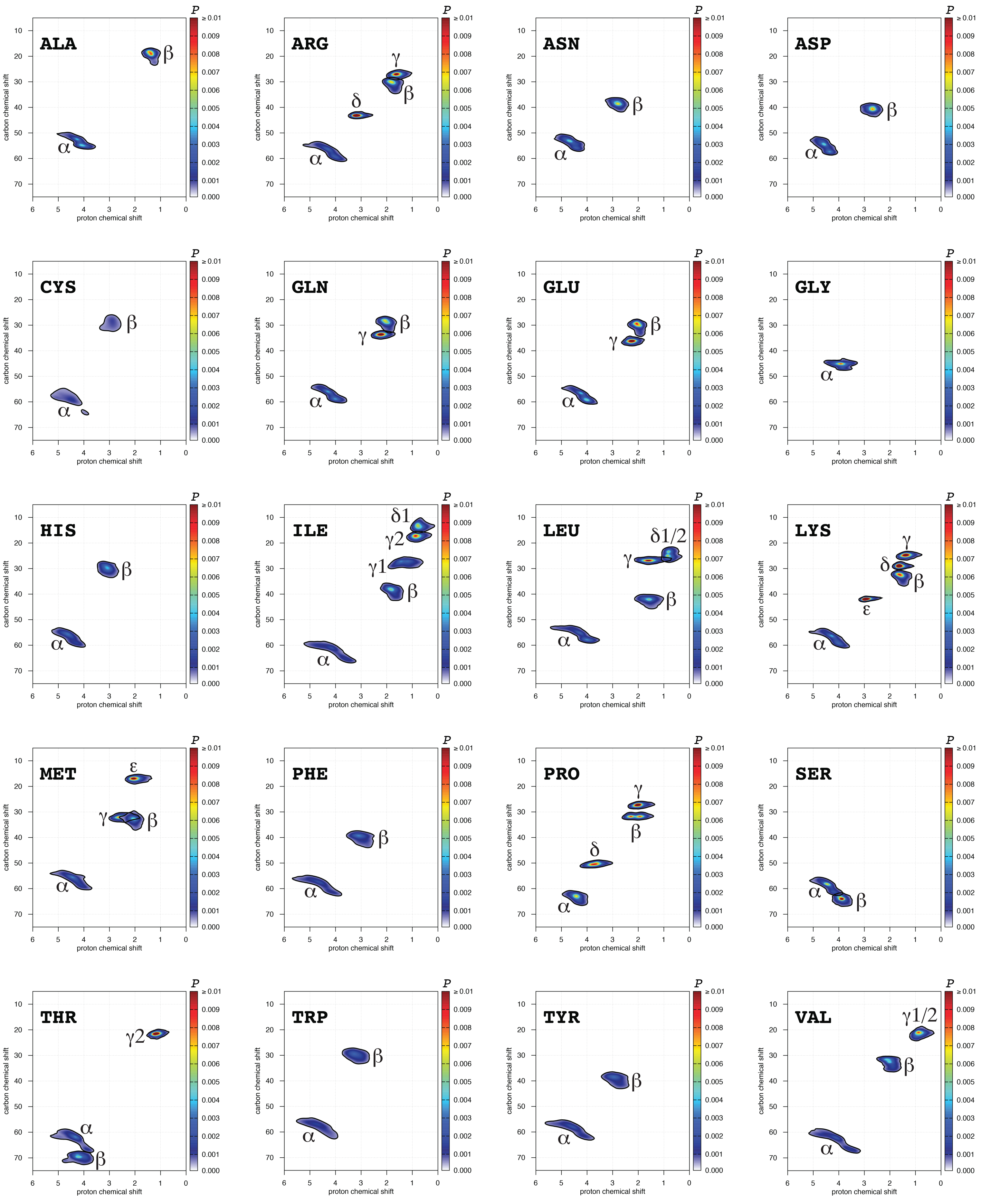

Supplementary Figure 2. Probability density maps of correlated aliphatic chemical shifts per amino acid. The color gradient is plotted with linear scaling, with black contours at a probability of 0.001. Probabilities smaller than the contour line are omitted for visualization purposes. Each map is labeled with Greek letters according to the ^{13}C-^{1}H moiety it refers to in the respective amino acid. Notice that the methyl groups of Leu and Val are indistinguishable and merged to a single map.

Supplementary Figure 3

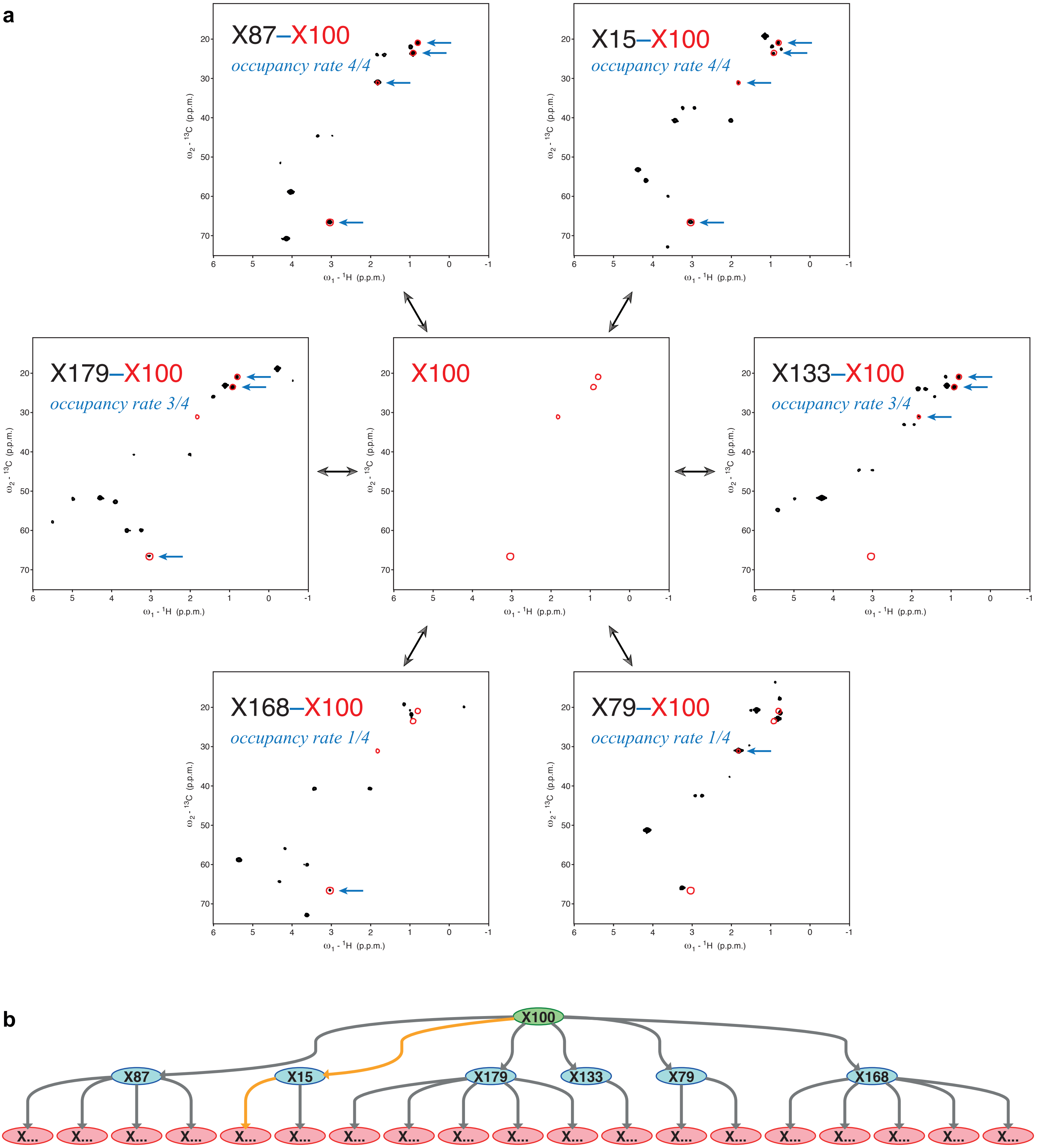

Supplementary Figure 3. Sequential connectivities established by matching correlated ^{13}C-^{1}H frequencies between the 4D-HCNH TOCSY and the 4D-HCNH NOESY spectra to generate chains of AAIGs, here denoted by X. (**a**) For every TOCSY AAIG (red, center) the correlated ^{13}C-^{1}H frequencies (i-1) are matched to the correlated ^{13}C-^{1}H frequencies (i and others) of any other NOESY AAIG (black). The occupancy rate of matched frequencies differs among the NOESY AAIGs (blue arrows). The higher the occupancy rate the more likely the sequential connectivity to be correct. For visualization purposes the carbon frequencies of folded peaks have been unfolded manually. (**b**) The connectivity information is used to generate a directed rooted tree from AAIG X100, here shown for a chain length of 3. The orange directed edges represent the correct path in the sequential assignment problem.

Supplementary Figure 4

RTT: 134 residues

TOCSY & NOESY (100.0%)

S	E	Q	F	T	T	K	L	N	T	L	E	D	S	Q	E	S	I	S	S	A	S	K	W	L
L	L	Q	Y	R	D	A	P	K	V	A	E	M	W	K	E	Y	M	L	P	R	S	V	N	T
R	R	K	L	L	G	L	Y	L	M	N	H	V	V	Q	Q	A	K	G	Q	K	I	I	Q	F
Q	D	S	F	G	K	V	A	A	E	V	L	G	R	I	N	Q	E	F	P	R	D	L	K	K
K	L	S	R	V	V	N	I	L	K	E	R	N	I	F	S	K	Q	V	V	N	D	I	E	E
R	S	L	A	A	A	L	E	H																

CBHBCAHA(CO)NH & NOESY (98.4%)

S	E	Q	F	T	T	K	L	N	T	L	E	D	S	Q	E	S	I	S	S	A	S	K	W	L
L	L	Q	Y	R	D	A	P	K	V	A	E	M	W	K	E	Y	M	L	P	R	S	V	N	T
R	R	K	L	L	G	L	Y	L	M	N	H	V	V	Q	Q	A	K	G	Q	K	I	I	Q	F
Q	D	S	F	G	K	V	A	A	E	V	L	G	R	I	N	Q	E	F	P	R	D	L	K	K
K	L	S	R	V	V	N	I	L	K	E	R	N	I	F	S	K	Q	V	V	N	D	I	E	E
R	S	L	A	A	A	L	E	H																

ms6282: 145 residues

TOCSY & NOESY (99.2%)

M	G	Q	V	S	A	V	S	T	V	L	I	N	A	E	P	A	A	V	L	A	A	I	S	D
Y	Q	T	V	R	P	K	I	L	S	S	H	Y	S	G	Y	Q	V	L	E	G	G	Q	G	A
G	T	V	A	T	W	K	L	Q	A	T	K	S	R	V	R	D	V	K	A	T	V	D	V	A
G	H	T	V	I	E	K	D	A	N	S	S	L	V	S	N	W	T	V	A	P	A	G	T	G
S	S	V	N	L	K	T	T	W	T	G	A	G	G	V	K	G	F	F	E	K	T	F	A	P
L	G	L	R	R	I	Q	D	E	V	L	E	N	L	K	K	H	V	E	G					

CBHBCAHA(CO)NH & NOESY (97.1%)

M	G	Q	V	S	A	V	S	T	V	L	I	N	A	E	P	A	A	V	L	A	A	I	S	D
Y	Q	T	V	R	P	K	I	L	S	S	H	Y	S	G	Y	Q	V	L	E	G	G	Q	G	A
G	T	V	A	T	W	K	L	Q	A	T	K	S	R	V	R	D	V	K	A	T	V	D	V	A
G	H	T	V	I	E	K	D	A	N	S	S	L	V	S	N	W	T	V	A	P	A	G	T	G
S	S	V	N	L	K	T	T	W	T	G	A	G	G	V	K	G	F	F	E	K	T	F	A	P
L	G	L	R	R	I	Q	D	E	V	L	E	N	L	K	K	H	V	E	G					

aLP: 198 residues

TOCSY & NOESY (96.3%)

A	N	I	V	G	G	I	E	Y	S	I	N	N	A	S	L	C	S	V	G	F	S	V	T	R
G	A	T	K	G	F	V	T	A	G	H	C	G	T	V	N	A	T	A	R	I	G	G	A	V
V	G	T	F	A	A	R	V	F	P	G	N	D	R	A	W	V	S	L	T	S	A	Q	T	L
L	P	R	V	A	N	G	S	S	F	V	T	V	R	G	S	T	E	A	A	V	G	A	A	V
C	R	S	G	R	T	T	G	Y	Q	C	G	T	I	T	A	K	N	V	T	A	N	Y	A	E
G	A	V	R	G	L	T	Q	G	N	A	C	M	G	R	G	D	S	G	G	S	W	I	T	S
A	G	Q	A	Q	G	V	M	S	G	G	N	V	Q	S	N	G	N	N	C	G	I	P	A	S
Q	R	S	S	L	F	E	R	L	Q	P	I	L	S	Q	Y	G	L	S	L	V	T	G		

CBHBCAHA(CO)NH & NOESY (95.3%)

A	N	I	V	G	G	I	E	Y	S	I	N	N	A	S	L	C	S	V	G	F	S	V	T	R
G	A	T	K	G	F	V	T	A	G	H	C	G	T	V	N	A	T	A	R	I	G	G	A	V
V	G	T	F	A	A	R	V	F	P	G	N	D	R	A	W	V	S	L	T	S	A	Q	T	L
L	P	R	V	A	N	G	S	S	F	V	T	V	R	G	S	T	E	A	A	V	G	A	A	V
C	R	S	G	R	T	T	G	Y	Q	C	G	T	I	T	A	K	N	V	T	A	N	Y	A	E
G	A	V	R	G	L	T	Q	G	N	A	C	M	G	R	G	D	S	G	G	S	W	I	T	S
A	G	Q	A	Q	G	V	M	S	G	G	N	V	Q	S	N	G	N	N	C	G	I	P	A	S
Q	R	S	S	L	F	E	R	L	Q	P	I	L	S	Q	Y	G	L	S	L	V	T	G		

nEIt: 248 residues

TOCSY & NOESY (99.6%)

M	L	K	G	V	A	A	S	P	G	I	A	I	G	K	A	F	L	Y	T	K	E	K	V	T
I	N	V	E	K	I	E	E	S	K	V	E	E	E	I	A	K	F	R	K	A	L	E	V	T
Q	E	E	I	E	K	I	K	E	K	A	L	K	E	F	G	K	E	K	A	E	I	F	E	A
H	L	M	L	A	S	D	P	E	L	I	E	G	V	E	N	M	I	K	T	E	L	V	T	A
D	N	A	V	N	K	V	I	E	Q	N	A	S	V	M	E	S	L	N	D	E	Y	L	K	E
R	A	V	D	L	R	D	V	G	N	R	I	I	E	N	L	L	G	V	K	S	V	N	L	S
D	L	E	E	E	V	V	V	I	A	R	D	L	T	P	S	D	T	A	T	M	K	K	E	M
V	L	G	F	A	T	D	V	G	G	R	T	S	H	T	A	I	M	A	R	S	L	E	I	P
A	V	V	G	L	G	N	V	T	S	Q	V	K	A	G	D	L	V	I	V	D	G	L	E	G
I	V	I	V	N	P	D	E	K	T	V	E	D	Y	K	S	K	K	E	S	Y	E	K		

CBHBCAHA(CO)NH & NOESY (99.6%)

M	L	K	G	V	A	A	S	P	G	I	A	I	G	K	A	F	L	Y	T	K	E	K	V	T
I	N	V	E	K	I	E	E	S	K	V	E	E	E	I	A	K	F	R	K	A	L	E	V	T
Q	E	E	I	E	K	I	K	E	K	A	L	K	E	F	G	K	E	K	A	E	I	F	E	A
H	L	M	L	A	S	D	P	E	L	I	E	G	V	E	N	M	I	K	T	E	L	V	T	A
D	N	A	V	N	K	V	I	E	Q	N	A	S	V	M	E	S	L	N	D	E	Y	L	K	E
R	A	V	D	L	R	D	V	G	N	R	I	I	E	N	L	L	G	V	K	S	V	N	L	S
D	L	E	E	E	V	V	V	I	A	R	D	L	T	P	S	D	T	A	T	M	K	K	E	M
V	L	G	F	A	T	D	V	G	G	R	T	S	H	T	A	I	M	A	R	S	L	E	I	P
A	V	V	G	L	G	N	V	T	S	Q	V	K	A	G	D	L	V	I	V	D	G	L	E	G
I	V	I	V	N	P	D	E	K	T	V	E	D	Y	K	S	K	K	E	S	Y	E	K		

Supplementary Figure 4. NH-mapping performance of 4D-CHAINS for four different protein targets. (left) 4D-CHAINS original implementation using a combination of 4D-HCNH TOCSY and 4D-HCNH NOESY spectra. (right) Synthetic data of a 4D CBHBCAHA(CO)NH experiment in combination with the 4D-HCNH NOESY spectrum. Mapping coverage is expressed as percentage of all assignable amide signals. Residue colouring: blue; proline, red; exchangeable amides, green; no TOCSY peaks observed. Assigned residues in salmon background, not assigned residues in white background, additionally not-assigned residues with the synthetic data in grey background.

Supplementary Figure 5

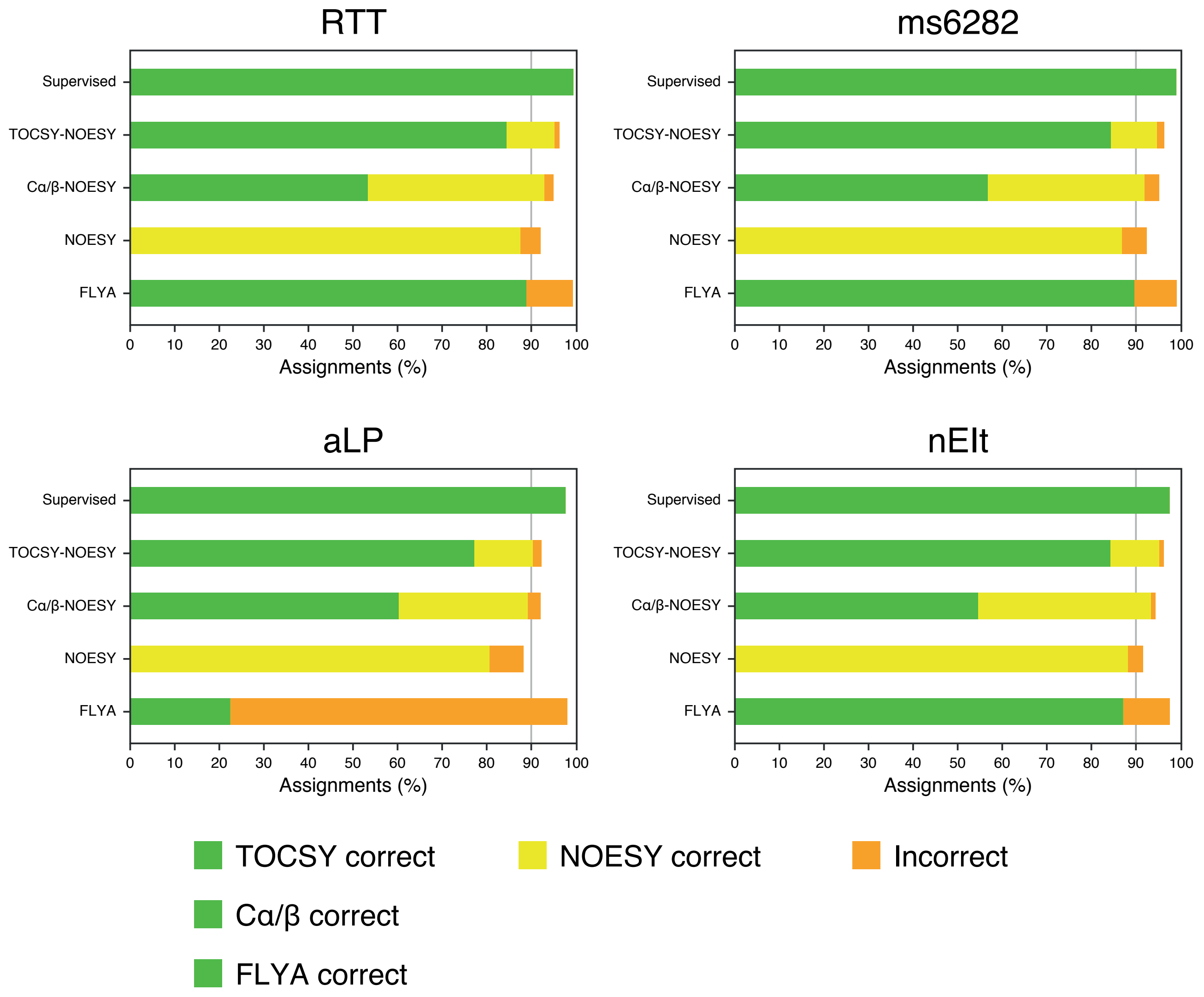

Supplementary Figure 5. Assignment statistics for all protein targets using 4D-CHAINS or FLYA. Quality of assignments using 4D-CHAINS for TOCSY-NOESY, Cα/β-NOESY, and NOESY input data, or FLYA input data. Input data explanation for the algorithms: TOCSY-NOESY, perform NH-mapping and aliphatic assignments using 4D-HCNH TOCSY and 4D-HCNH NOESY; Cα/β-NOESY, perform NH-mapping and aliphatic assignments using 4D CBHBCAHA(CO)NH synthetic data and 4D-HCNH NOESY; NOESY, perform aliphatic assignments using fixed $^{1}H,^{15}N$ HSQC assignments and 4D-HCNH NOESY; FLYA, perform NH-mapping and aliphatic assignments using 4D-HCNH TOCSY, 4D-HCNH NOESY, and 4D-HCCH NOESY.

Supplementary Figure 6

a

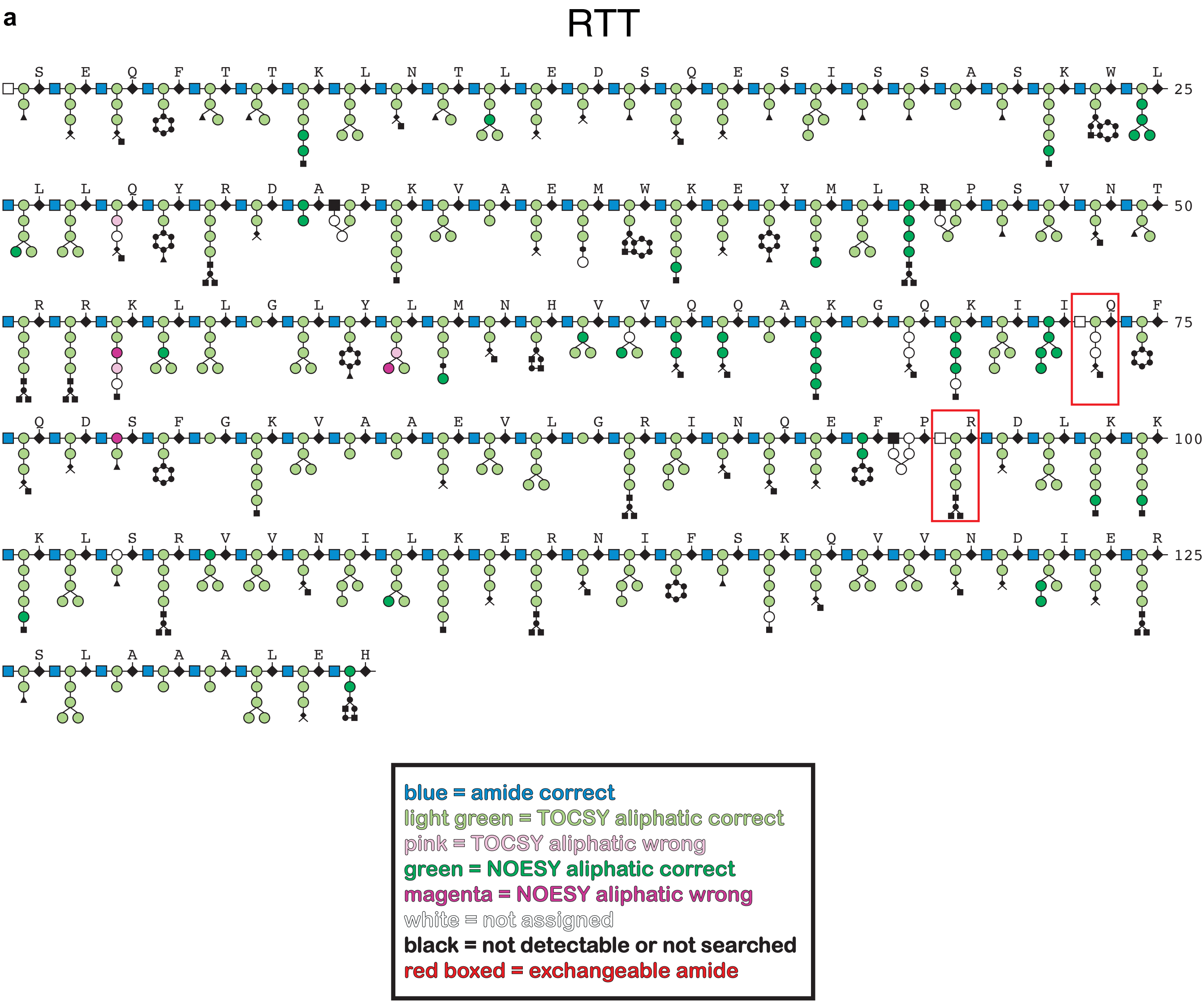

Supplementary Figure 6. Per atom assignment performance of 4D-CHAINS using a combination of 4D-HCNH TOCSY and 4D-HCNH NOESY spectra for each protein target. (**a**) RTT protein. 4D-CHAINS does not deal with assignments of aromatic groups of His, Phe, Tyr, or Trp residues, and of sidechain amide groups of Asn or Gln residues.

Supplementary Figure 6

b

ms6282

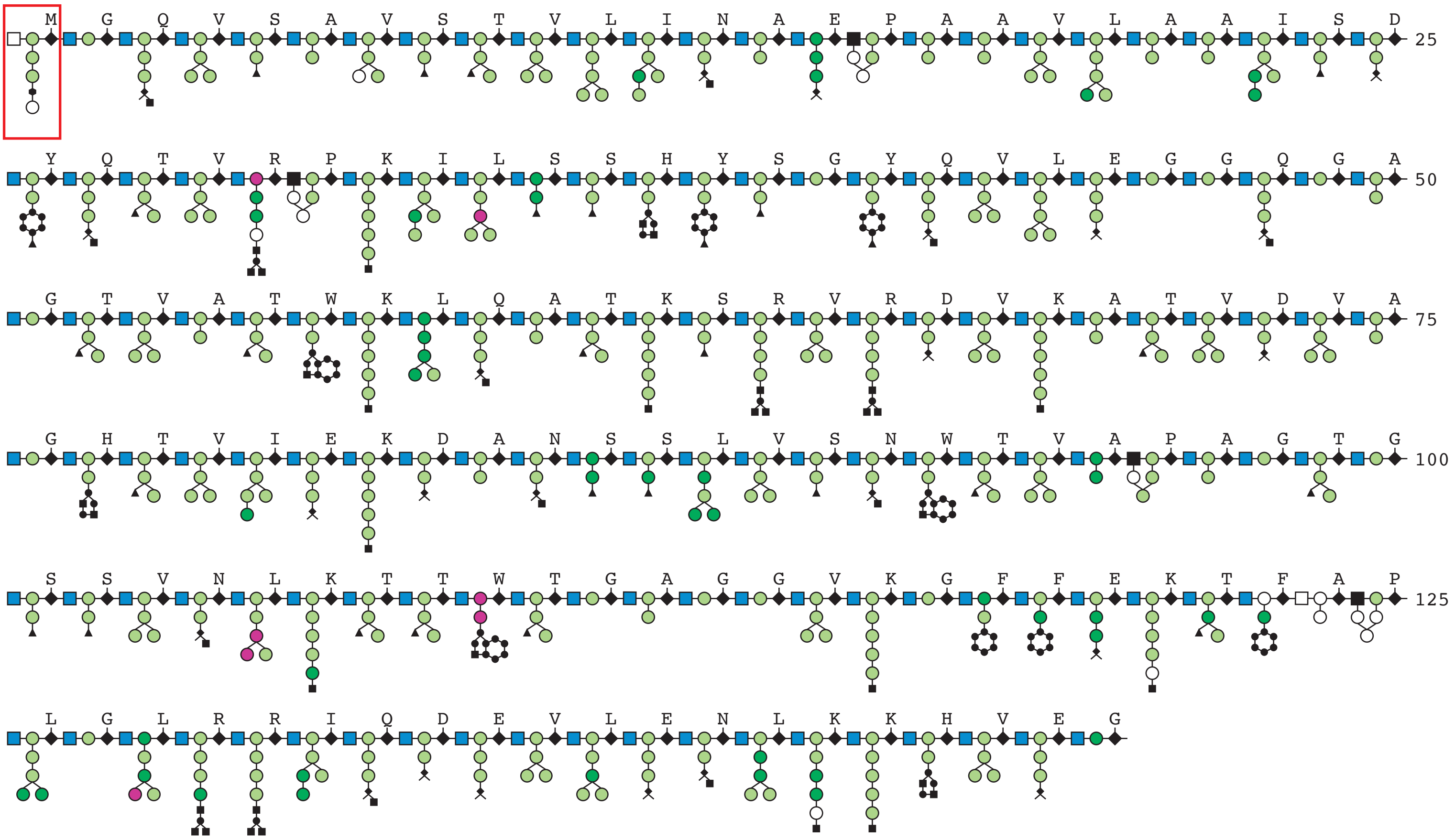

blue = amide correct
light green = TOCSY aliphatic correct
pink = TOCSY aliphatic wrong
green = NOESY aliphatic correct
magenta = NOESY aliphatic wrong
white = not assigned
black = not detectable or not searched
red boxed = exchangeable amide

Supplementary Figure 6. Per atom assignment performance of 4D-CHAINS using a combination of 4D-HCNH TOCSY and 4D-HCNH NOESY spectra for each protein target. (**b**) ms6282 protein. 4D-CHAINS does not deal with assignments of aromatic groups of His, Phe, Tyr, or Trp residues, and of sidechain amide groups of Asn or Gln residues.

Supplementary Figure 6

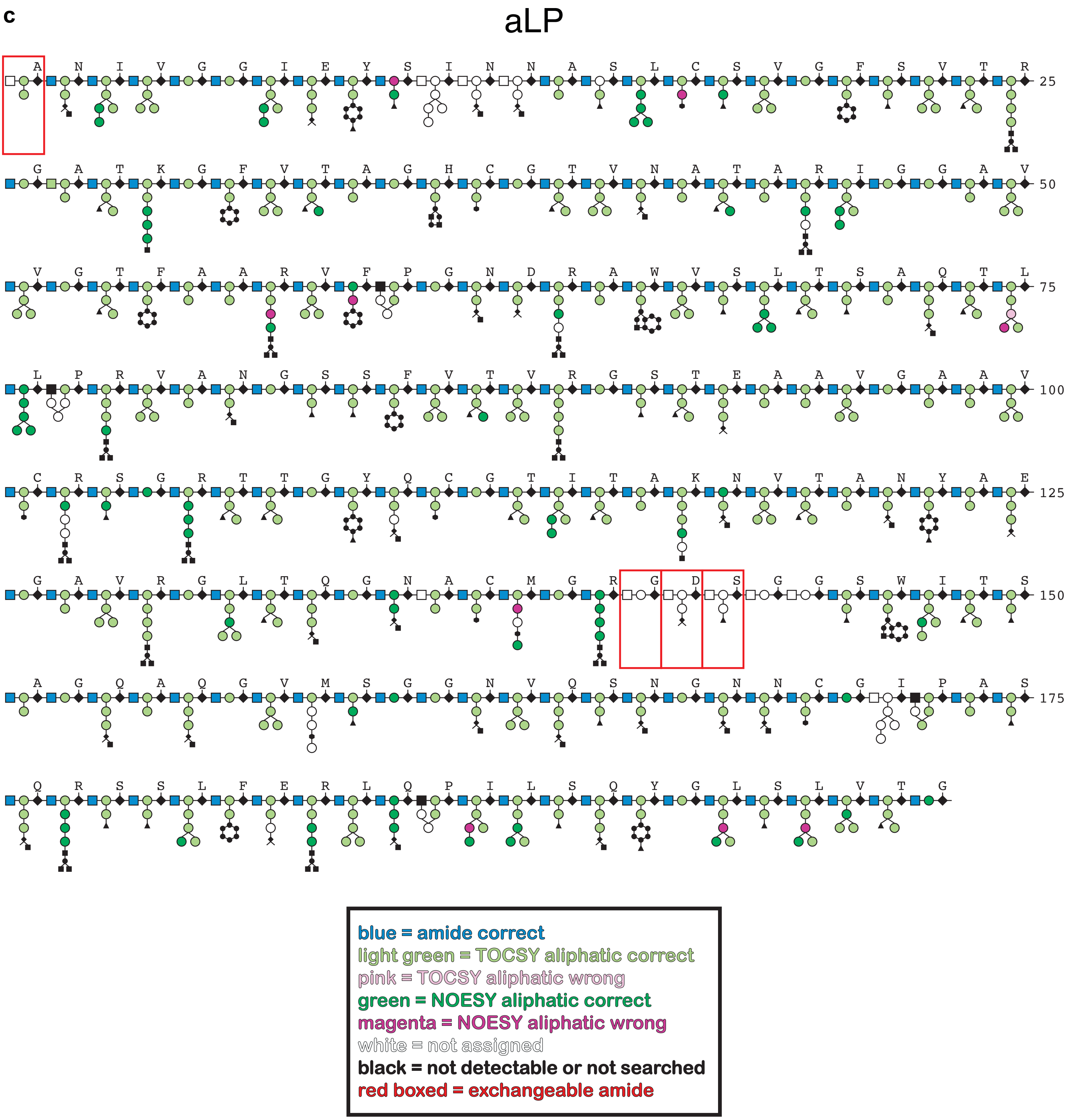

Supplementary Figure 6. Per atom assignment performance of 4D-CHAINS using a combination of 4D-HCNH TOCSY and 4D-HCNH NOESY spectra for each protein target. (**c**) aLP protein. 4D-CHAINS does not deal with assignments of aromatic groups of His, Phe, Tyr, or Trp residues, and of sidechain amide groups of Asn or Gln residues.

Supplementary Figure 6

d

nEIt

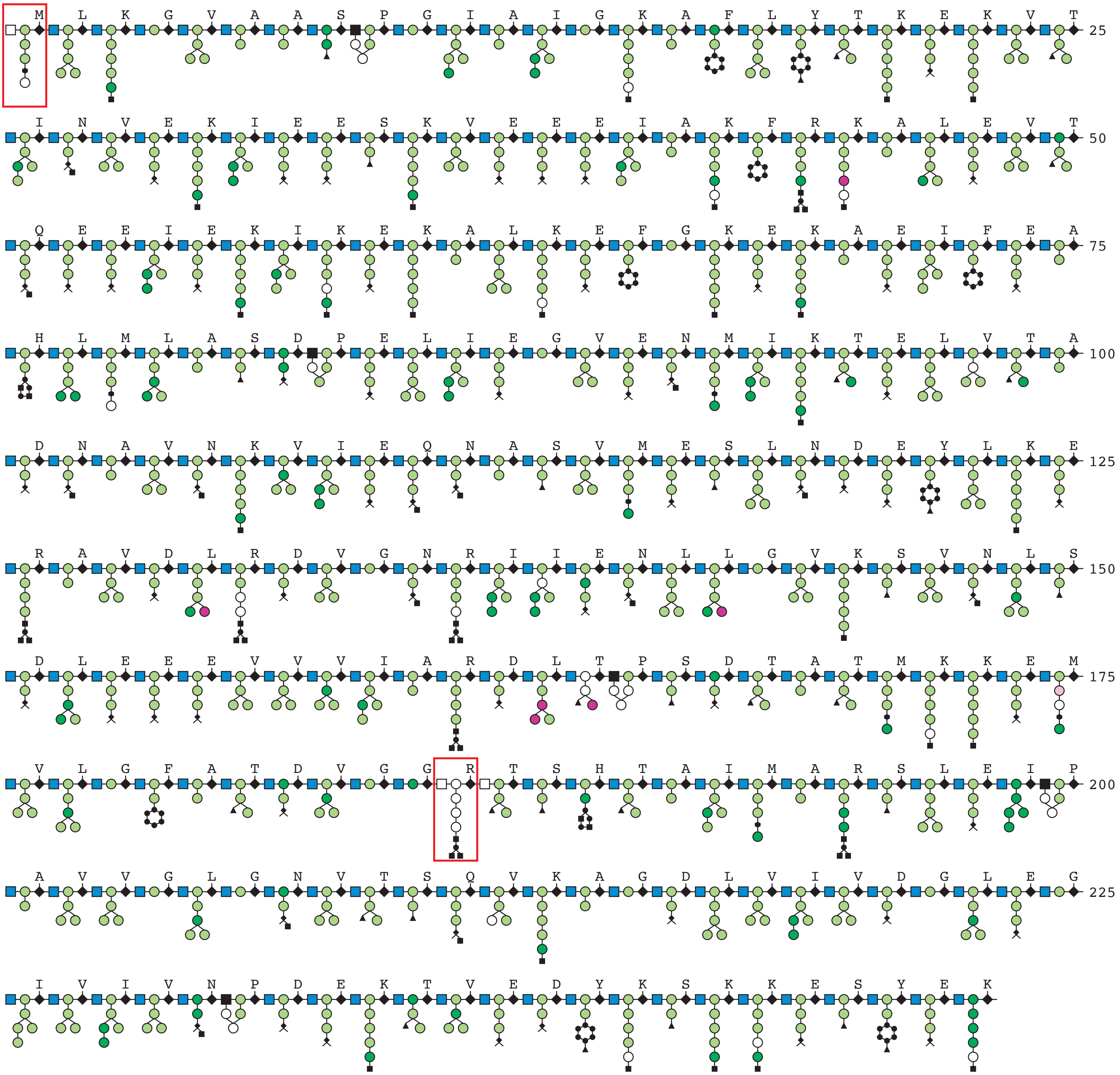

blue = amide correct
light green = TOCSY aliphatic correct
pink = TOCSY aliphatic wrong
green = NOESY aliphatic correct
magenta = NOESY aliphatic wrong
white = not assigned
black = not detectable or not searched
red boxed = exchangeable amide

Supplementary Figure 6. Per atom assignment performance of 4D-CHAINS using a combination of 4D-HCNH TOCSY and 4D-HCNH NOESY spectra for each protein target. (**d**) nEIt protein. 4D-CHAINS does not deal with assignments of aromatic groups of His, Phe, Tyr, or Trp residues, and of sidechain amide groups of Asn or Gln residues.

Supplementary Figure 7

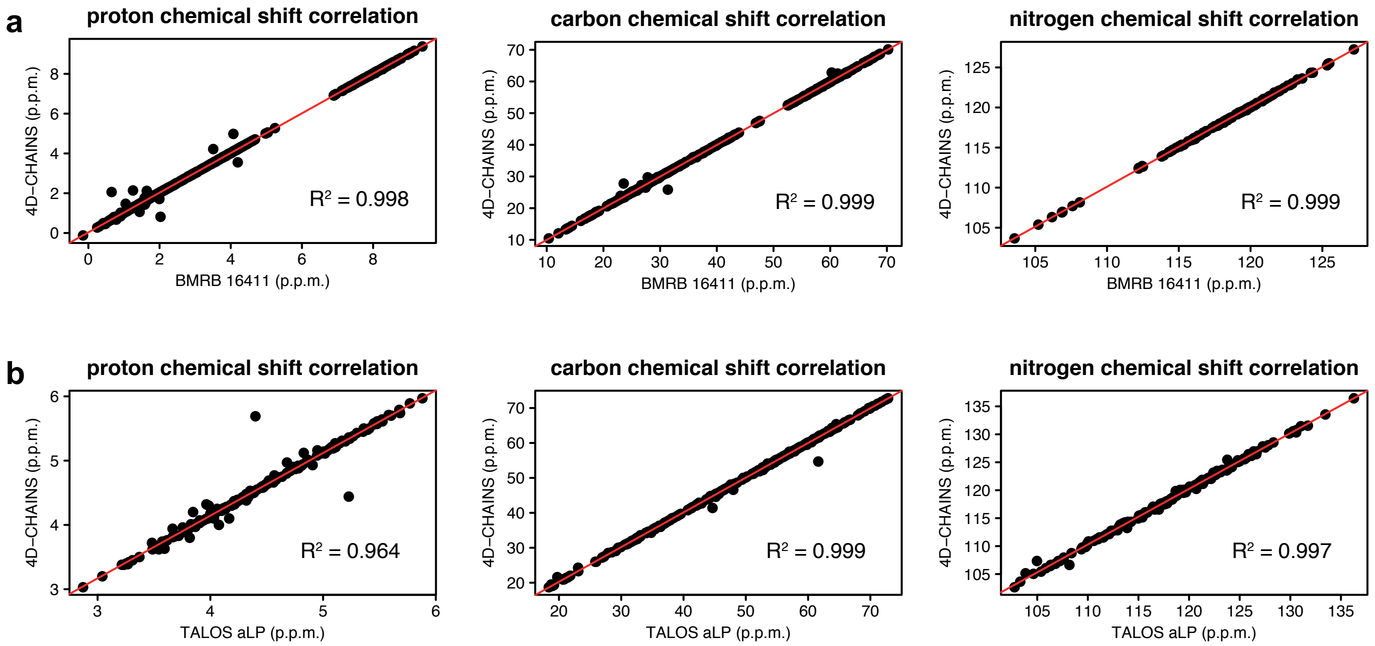

Supplementary Figure 7. Correlation of 4D-CHAINS fully automated chemical shift assignments and chemical shifts deposited to databases obtained by conventional NMR methods. (**a**) For RTT the complete set of assignments is available under BMRB ID 16411. (**b**) For aLP only backbone chemical shifts (N, C^{α}, C^{β}, H^{α}, H^{β}) are available from the TALOS library. For aLP the NMR experiments listed in the original publication were recorded at 35 °C whereas the 4D spectra of the present study were recorded at 25 °C.

Supplementary Figure 8

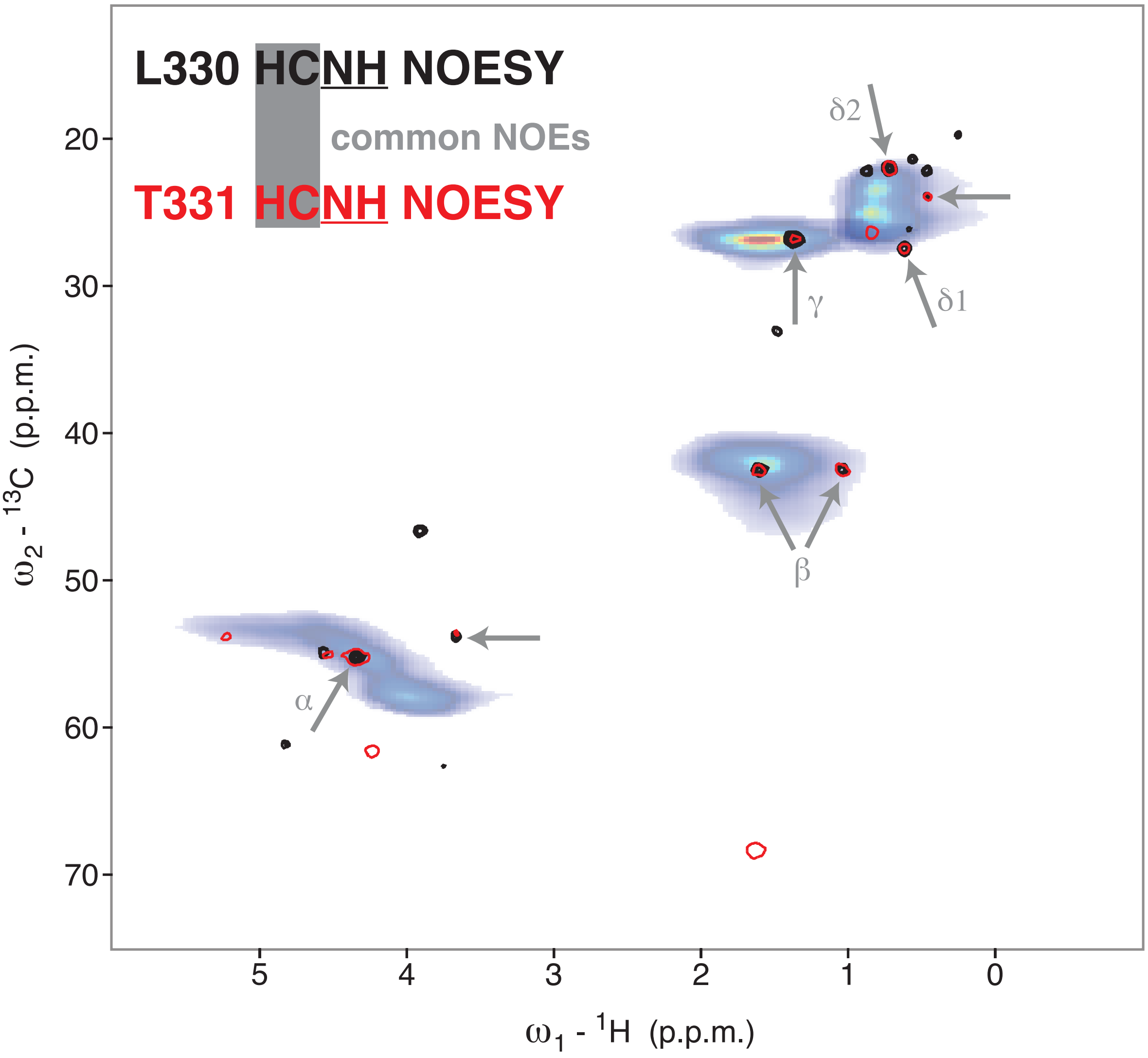

Supplementary Figure 8. 4D-CHAINS employs the concept of common NOEs between successive amides in order to derive assignments of aliphatic atoms from the 4D-HCNH NOESY spectrum. Based on fixed ^{15}N-^{1}H frequencies 4D-CHAINS has clustered 18 NOE peaks belonging to L330 amide (black) and 13 NOE peaks belonging to T331 amide (red). The question in place is to assign the ^{13}C-^{1}H aliphatic frequencies of L330. To do so, 4D-CHAINS first identifies the common NOEs L330 amide shares with the next amide in protein sequence, that is T331. The total number of common NOEs is 8, indicated by gray arrows. For the common NOEs, 4D-CHAINS derives probabilties for each of them belonging to certain atom types of Leu from the 2D probability map, shown in the background. Each probability is modified by the intensity of the corresponding peak to a score. The highest product of scores provides the assignments for the missing atom types. In this case all atom types are assigned correctly (labeled arrows). For visualization purposes the carbon frequencies of folded peaks have been unfolded manually.

Supplementary Figure 9

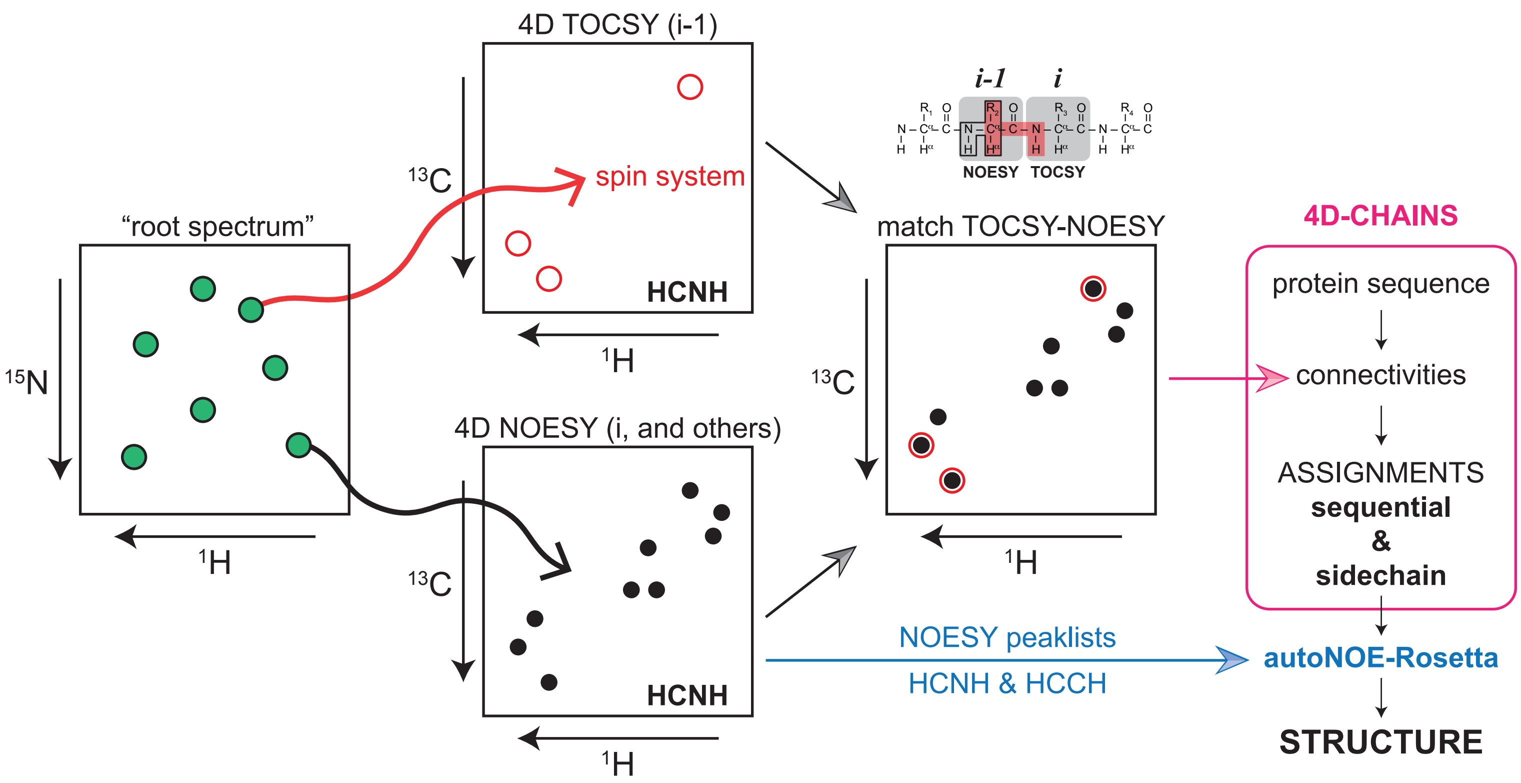

Supplementary Figure 9. Schematic overview of the novel, fully automated, approach to NMR assignments and structure of large protonated proteins. The 4D-HCNH TOCSY spectrum allows for spin system identification by coupling the preceding aliphatic ^{13}C-^{1}H resonances to the backbone amide. The 4D-HCNH NOESY spectrum, on the other hand, reports through-space correlations for a backbone amide, including mainly the intraresidue ones. The novel algorithm 4D-CHAINS uses 2D probability density maps of chemical shifts to first identify the spin systems, and then match spin system information between the two spectra (TOCSY-NOESY). The advantage is that the correlated chemical shifts of ^{13}C-^{1}H moieties establish robust connectivities for resonance assignment in one shot (sequential and sidechain) and reduce ambiguities in downstream NOE-based structure calculation. 4D-CHAINS automated assignments are then passed to autoNOE-Rosetta, together with the peaklists of two 4D NOESY spectra (HCNH and HCCH), for unsupervised NMR structure determination of large protonated proteins.

Supplementary Figure 10

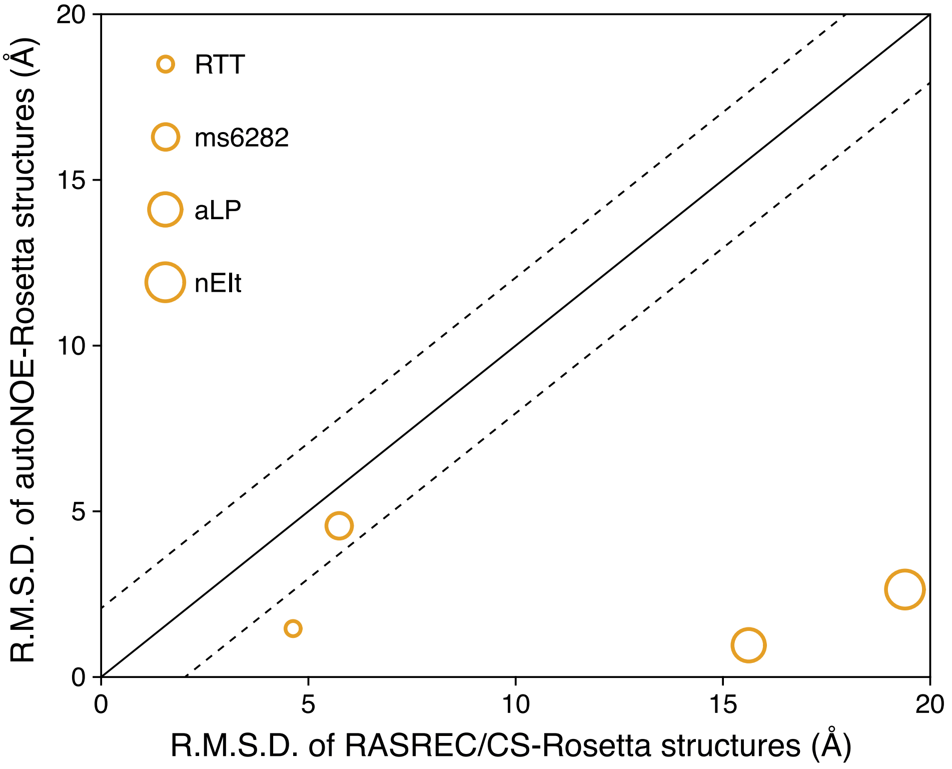

Supplementary Figure 10. Comparison of RASREC/CS-Rosetta and autoNOE-Rosetta ensembles. Average R.M.S.D. values of 10 lowest energy structures obtained from RASREC/CS-Rosetta and autoNOE-Rosetta structure calculations, compared to the closest PDB deposited reference structures. The reference structures used for RTT is a solution NMR structure with 100% sequence identity (PDB ID 2KM), for ms6282 is a crystal structure with 30% sequence identity (PDB ID 4PSB), for aLP is a crystal structure with 100% sequence identity (PDB ID 1P01) and for nElt is a solution NMR structure with 45% sequence identity (PDB ID 1EZB). Diagonal line indicates equal R.M.S.D. values of ensembles calculated using the two methods. Adjacent dashed lines indicate R.M.S.D. values of RASREC/CS-Rosetta and autoNOE-Rosetta ensembles within 2Å from one another. Points below the diagonal correspond to lower R.M.S.D. values for ensembles calculated using autoNOE-Rosetta relative to RASREC/CS-Rosetta. The area of the circles in the plot is proportional to the size of proteins (number of residues). The autoNOE-Rosetta ensembles were calculated using supervised resonance assignments and both HCNH+HCCH NOE peak lists.

Supplementary Figure 11

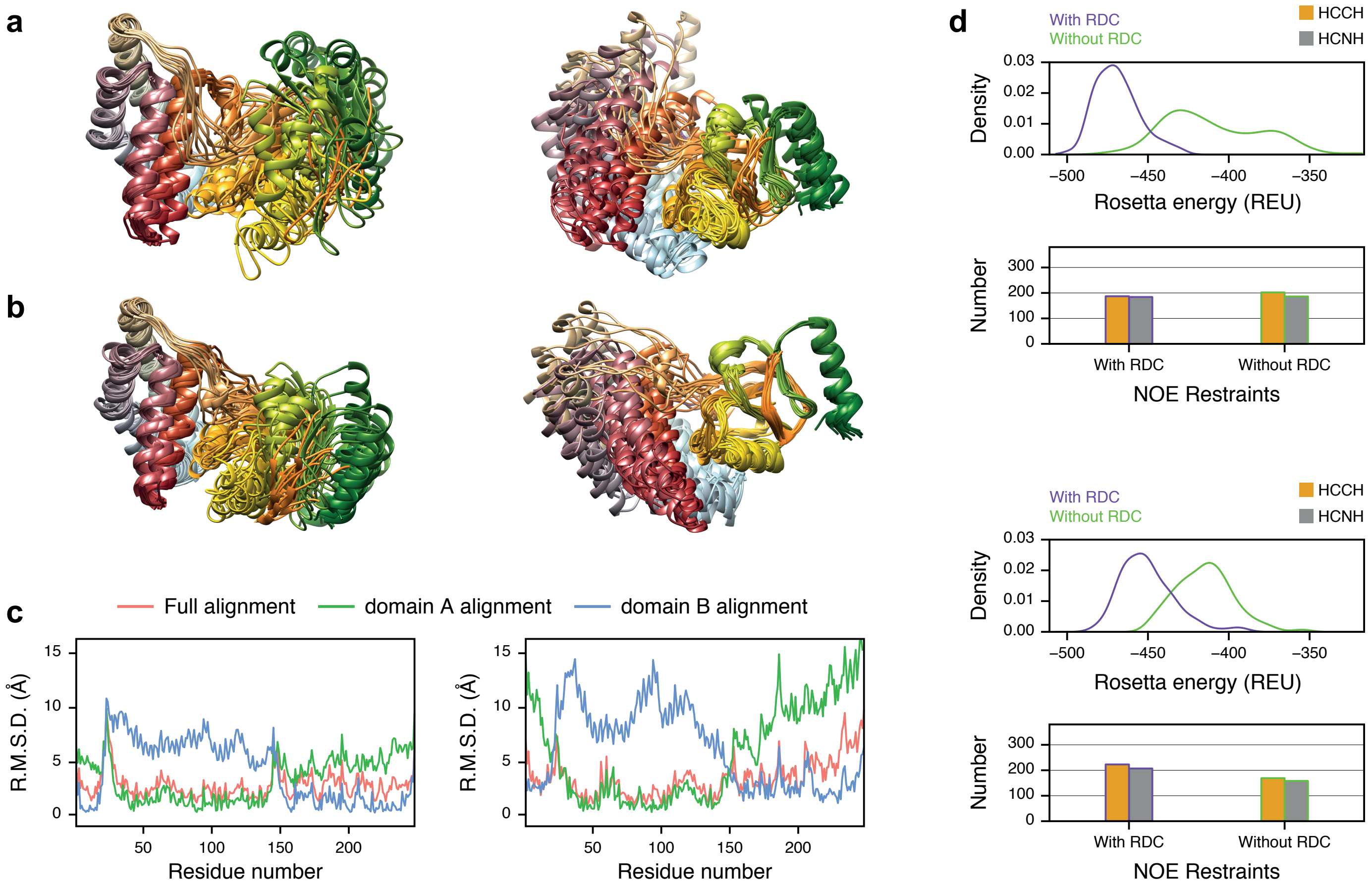

Supplementary Figure 11. Enzyme I structures generated using chemical shift and NOE data. (**a**) Enzyme I structures calculated using supervised assignments and HCNH+HCCH NOEs. Left: 10 lowest-energy structures superimposed with respect to domain A. Right: 10 lowest-energy structures superimposed with respect to domain B. (**b**) Enzyme I structures calculated using automated 4D-CHAINS assignments (derived from TOCSY-NOESY spectra) and HCNH+HCCH NOEs. Left: 10 lowest-energy structures superimposed with respect to domain A. Right: 10 lowest-energy structures superimposed with respect to domain B. (**c**) Average R.M.S.D. per residue among 10 lowest-energy structures aligned with respect to different domain selections. Left: R.M.S.D. values of structures sampled using supervised assignments. Right: R.M.S.D. values of structures sampled using 4D-CHAINS automated assignments. Full alignment (crimson): Global alignment of the 10 lowest-energy structures. Domain A alignment (dark green): Alignment of the 10 lowest-energy structures over residues 1-143. Domain B alignment (blue): Alignment of the 10 lowest-energy structures over residues 144-248. (**d**) Rosetta energy (in REU, Rosetta Energy Units) distributions of 100 lowest-energy structures sampled during the final stage of autoNOE-Rosetta calculations with (purple) and without (green) using RDCs, in addition to chemical shifts and NOEs. The bars indicate the total numbers of assigned long range HCNH (amide to aliphatic) and HCCH (aliphatic to aliphatic) restraints, including ambiguous restraints obtained for different stereo-specific groups. Images of structural ensembles were produced using Chimera (https://www.cgl.ucsf.edu/chimera).

Supplementary Figure 12

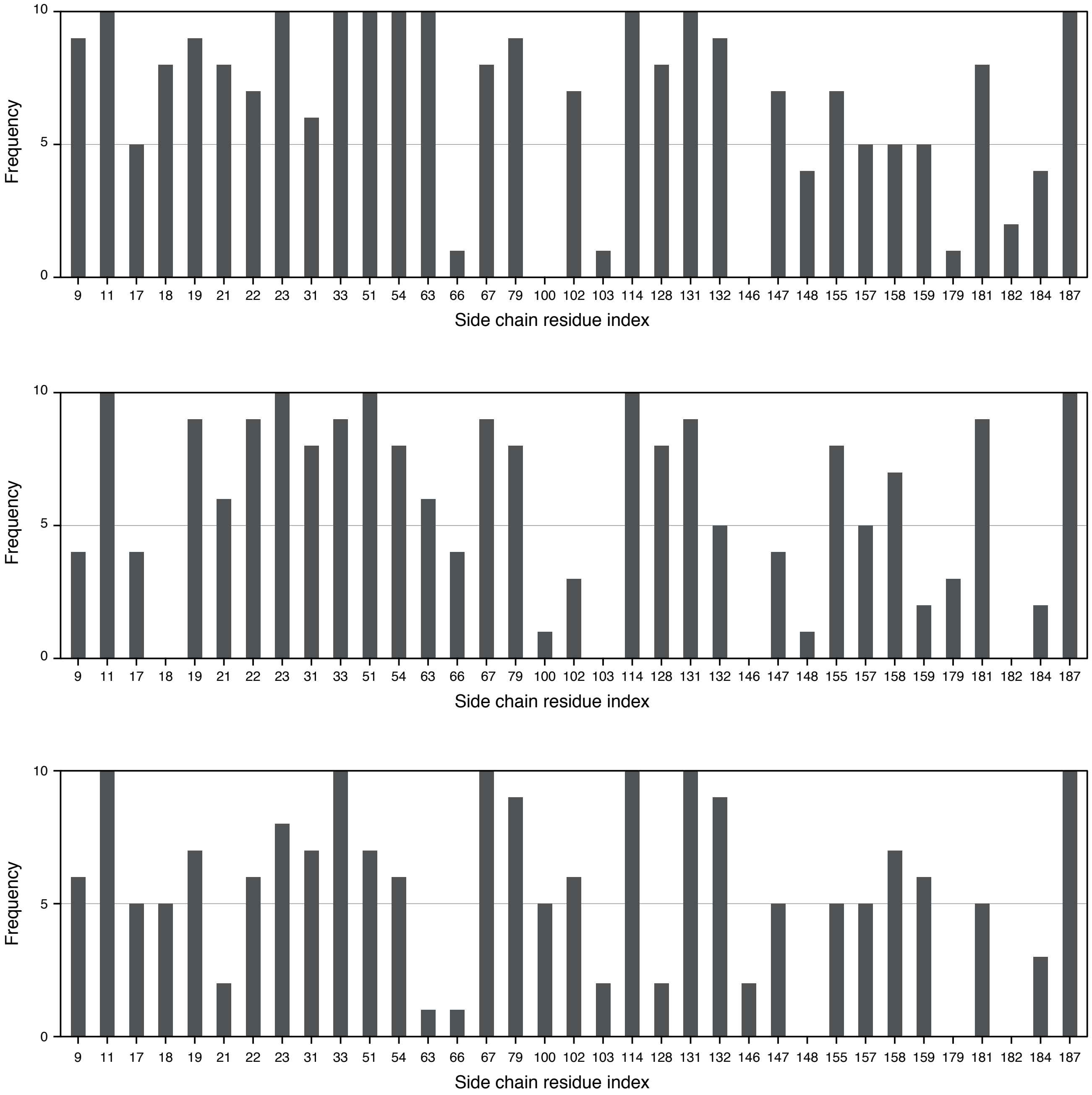

Supplementary Figure 12. Frequency of the buried residues in 10 lowest-energy structures with correct χ^1 dihedral placements. All the panels represent frequencies of the residues in 10 lowest-energy models, whose dihedral placements (χ^1) are within 30° (considered correct) from the corresponding rotamers in the X-ray structure. The buried residues were calculated using 10 Å^2 solvent accessible surface area threshold and further filtered based on secondary structure (α-helix and β-sheet) to retain only the core, rigid residues. Of the core, rigid residues only those residues were used whose side chain conformers were consistent with crystallographic ensemble of 6 structures (PDB IDs 1P01, 1QRX, 1SSX, 1TAL, 2ALP, 2H5C and 2ULL). The top panel was obtained for the structural ensemble calculated using supervised assignments with HCNH+HCCH NOEs. The middle and the bottom panels were obtained for the structural ensembles calculated using 4D-CHAINS TOCSY-NOESY automated assignments with HCNH and HCNH+HCCH NOEs. Solvent accessible surface area was calculated using PyMOL software (https://www.pymol.org).

Supplementary Figure 13

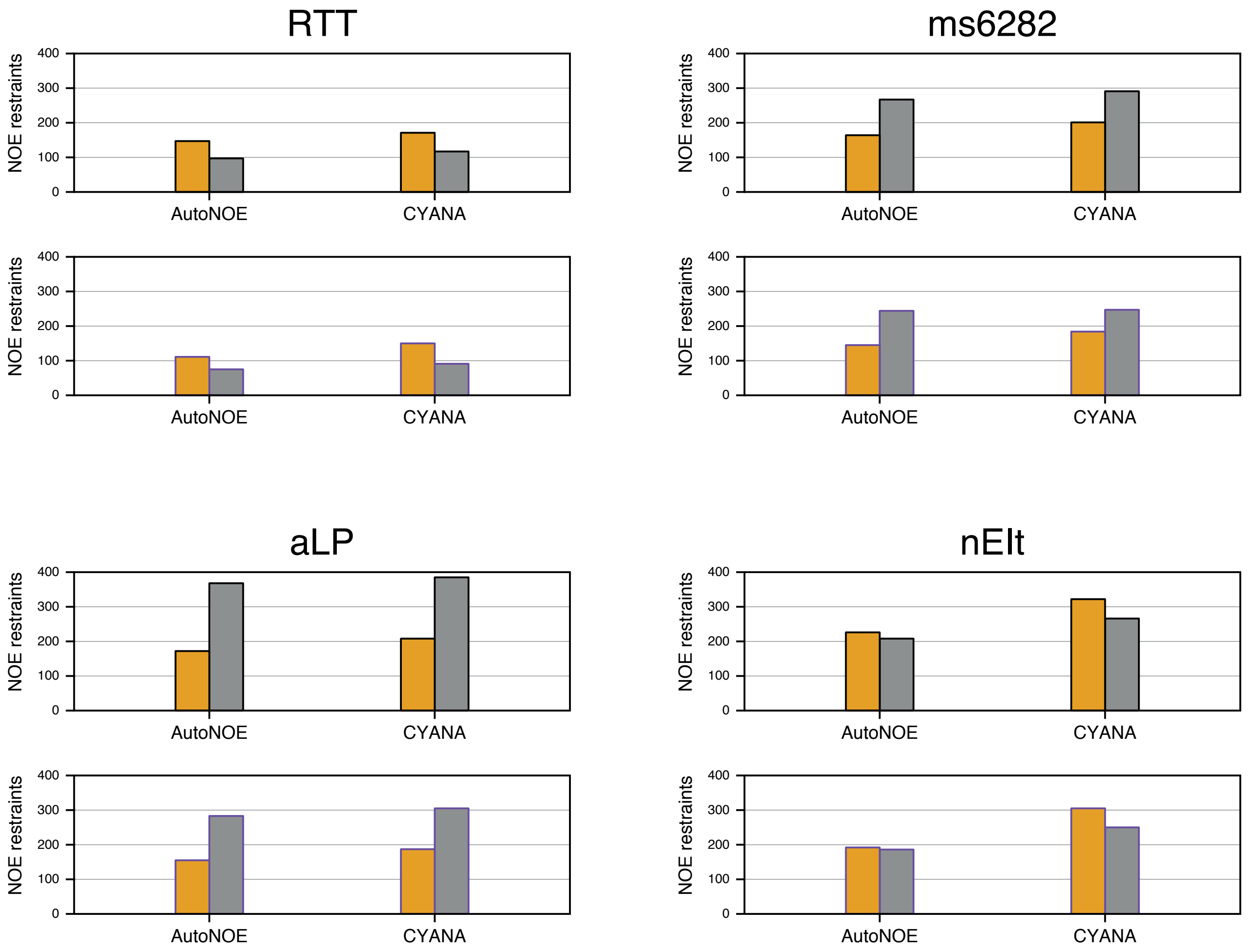

Supplementary Figure 13. Comparison of NOE restraints automatically assigned by autoNOE-Rosetta and CYANA . All the panels represent number of total long-range HCCH (orange) and HCNH (grey) NOE restraints predicted by autoNOE-Rosetta and CYANA using supervised assignments (black) and automated 4D-CHAINS assignments derived from TOCSY-NOESY spectra (purple).

Supplementary Figure 14

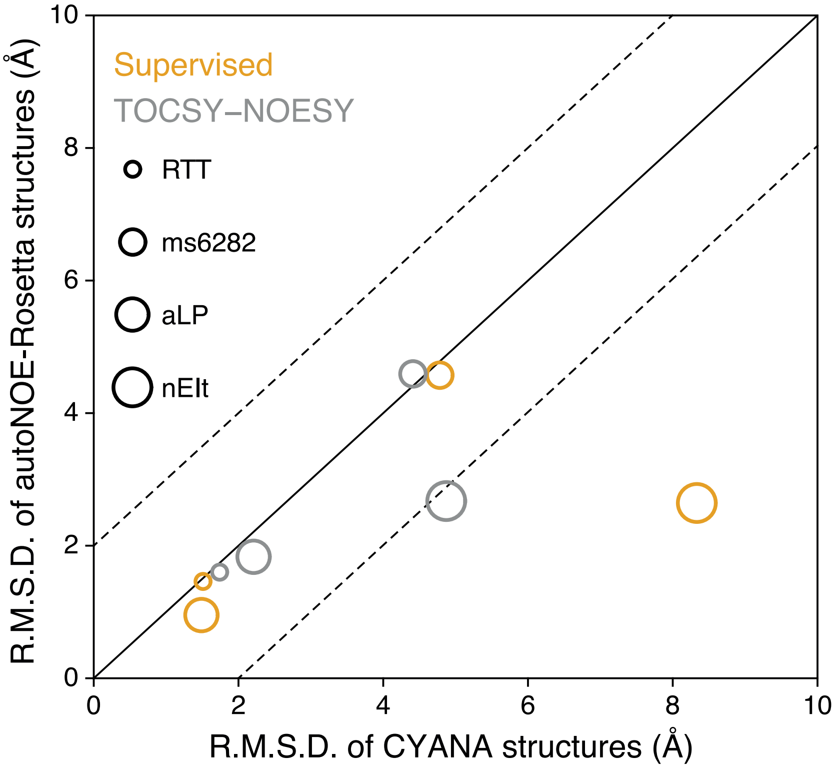

Supplementary Figure 14. Comparison of CYANA and autoNOE-Rosetta ensembles. Average R.M.S.D. values of 10 lowest-energy structural models obtained from autoNOE-Rosetta structure calculations and 10 structural models obtained from CYANA filtered using lowest target function, compared to the closest PDB deposited reference structures. The reference structures used for RTT is a solution NMR structure with 100% sequence identity (PDB ID 2KM), for ms6282 is a crystal structure with 30% sequence identity (PDB ID 4PSB), for aLP is a crystal structure with 100% sequence identity (PDB ID 1P01) and for nElt is a solution NMR structure with 45% sequence identity (PDB ID 1EZB). Diagonal line indicates equal R.M.S.D. values of ensembles calculated using the two methods. Adjacent dashed lines indicate R.M.S.D. values of CYANA and autoNOE-Rosetta ensembles within 2Å from one another. Points below the diagonal correspond to lower R.M.S.D. values for ensembles calculated using autoNOE-Rosetta relative to CYANA. The area of the circles in the plot is proportional to the size of proteins (number of residues). The autoNOE-Rosetta and CYANA ensembles were calculated using supervised (yellow) and automated (4D-CHAINS using TOCSY-NOESY spectra) (gray) resonance assignments and both HCNH+HCCH NOE peak lists.

Supplementary Table 1

Supplementary Table 1. 4D-CHAINS performance in obtaining NOESY-based assignments using the concept of common NOEs in different settings.

Using 2Dprob							
Setting	Missing	Assigned	Correct	Wrong	Error rate	Combined error rate	Completeness
TOCSY-NOESY	387	278	234	44	15.8%	2.3%	95.1%
Cα/β-NOESY	979	850	750	100	11.8%	4.8%	94.2%
NOESY	2232	2042	1788	254	12.4%	–	91.5%

Using 2Dprob $*$ (100 $*$ intensity^2)							
Setting	Missing	Assigned	Correct	Wrong	Error rate	Combined error rate	Completeness
TOCSY-NOESY	387	277	253	24	8.7%	1.3%	95.1%
Cα/β-NOESY	979	847	804	43	5.1%	2.0%	94.1%
NOESY	2232	2033	1921	112	5.5%	–	91.1%

Missing refers to aliphatic carbon types that could not be assigned from TOCSY or Cα/β synthetic data to yield 100% completeness of aliphatic chemical shifts for the four protein targets. For TOCSY-NOESY and Cα/β-NOESY, 4D-CHAINS performed correctly the mapping of backbone amide frequencies to the protein sequences, whereas for NOESY, the $^1H,^{15}N$ HSQC frequencies were fixed. 4D-CHAINS performance is shown when using only 2D probability heat maps or a combined function that takes into account the corresponding relative peak intensities in obtaining atom type assignments from the 4D-HCNH NOESY spectrum. Combined error rate corresponds to the overall 4D-CHAINS assignment performance using a combination of TOCSY-NOESY or Cα/β-NOESY.

Supplementary Table 2

Supplementary Table 2. Complete violation statistics for long-range restraints in the 10 lowest-energy models computed for targets RTT, ms6282, aLP and nElt.

Protein	NOE type‡	Total no. of NOEs*	Viol (< 1 Å)[1]	Viol (1 – 2 Å)[2]	Viol (2 – 5 Å)[3]	Viol (> 5 Å)[4]
RTT	All assigned	244	4.6 ± 1.8	0.6 ± 0.49	0.1 ± 0.3	0.0 ± 0.0
	HI_UNAMBIG	0	0.0 ± 0.0	0.0 ± 0.0	0.0 ± 0.0	0.0 ± 0.0
	MED_UNAMBIG	118	0.7 ± 0.64	0.0 ± 0.0	0.0 ± 0.0	0.0 ± 0.0
	LOW_AMBIG	126	3.9 ± 1.76	0.6 ± 0.49	0.1 ± 0.3	0.0 ± 0.0
	MED_AMBIG	0	0.0 ± 0.0	0.0 ± 0.0	0.0 ± 0.0	0.0 ± 0.0
ms6282	All assigned	431	16.8 ± 2.36	0.4 ± 0.66	0.0 ± 0.0	0.0 ± 0.0
	HI_UNAMBIG	2	0.0 ± 0.0	0.0 ± 0.0	0.0 ± 0.0	0.0 ± 0.0
	MED_UNAMBIG	207	5.5 ± 0.67	0.0 ± 0.0	0.0 ± 0.0	0.0 ± 0.0
	LOW_AMBIG	222	11.3 ± 2.69	0.4 ± 0.66	0.0 ± 0.0	0.0 ± 0.0
	MED_AMBIG	0	0.0 ± 0.0	0.0 ± 0.0	0.0 ± 0.0	0.0 ± 0.0
aLP	All assigned	540	21.6 ± 6.23	5.0 ± 1.67	1.4 ± 1.36	0.2 ± 0.4
	HI_UNAMBIG	3	0.1 ± 0.3	0.0 ± 0.0	0.0 ± 0.0	0.0 ± 0.0
	MED_UNAMBIG	181	7.4 ± 2.65	1.3 ± 1.35	0.1 ± 0.0	0.0 ± 0.0
	LOW_AMBIG	355	14.0 ± 4.86	3.6 ± 1.5	1.3 ± 1.1	0.2 ± 0.4
	MED_AMBIG	1	0.1 ± 0.3	0.1 ± 0.3	0.0 ± 0.0	0.0 ± 0.0
nElt	All assigned	378	4.4 ± 2.24	1.2 ± 1.08	0.4 ± 0.66	0.0 ± 0.0
	HI_UNAMBIG	3	0.1 ± 0.3	0.0 ± 0.0	0.0 ± 0.0	0.0 ± 0.0
	MED_UNAMBIG	147	1.6 ± 1.28	0.5 ± 0.92	0.4 ± 0.66	0.0 ± 0.0
	LOW_AMBIG	224	2.7 ± 1.35	0.7 ± 0.64	0.0 ± 0.0	0.0 ± 0.0
	MED_AMBIG	4	0.0 ± 0.0	0.0 ± 0.0	0.0 ± 0.0	0.0 ± 0.0

‡Classes of structurally degenerate NOE restraints based on confidence score as assigned by autoNOE-Rosetta.
*Total number of NOE restraints in each class
[1]Average number of distance violations within 1 Å
[2]Average number of distance violations between 1 – 2 Å
[3]Average number of distance violations between 2 – 5 Å
[4]Average number of distance violations greater than 5 Å

Supplementary Table 3

Supplementary Table 3. NOE contacts involving methyl groups identified by autoNOE-Rosetta for protein targets RTT, ms6282, aLP and nElt.

Protein target	Assignments	Total raw NOE contacts*	Methyl-Methyl NOE contacts~
RTT	Supervised	244	66
	TOCSY-NOESY	186	60
ms6282	Supervised	431	61
	TOCSY-NOESY	389	59
aLP	Supervised	540	108
	TOCSY-NOESY	438	97
nElt	Supervised	434	141
	TOCSY-NOESY	378	120

*Structurally degenerate HCNH+HCCH NOE contacts
~Structurally degenerate methyl-methyl NOE contacts

Supplementary Table 4

Supplementary Table 4. Assignments of methyl groups.

Protein	Expected	Supervised correct	4D-CHAINS correct/wrong	FLYA correct/wrong
RTT	82	82	80/1	68/14
ms6282	98	97	93/2	86/10
aLP	118	118	111/1	27/91
nEIt	177	175	170/4	156/18
All	475	472	454/8	337/133

By default, 4D-CHAINS automated assignments are derived from two spectra (4D-HCNH TOCSY, 4D-HCNH NOESY). FLYA automated assignments were produced using three spectra as input (4D-HCNH TOCSY, 4D-HCNH NOESY, 4D-HCCH NOESY).

Supplementary Table 5

Supplementary Table 5. Accuracy of buried core side chains of aLP relative to the X-ray structure.

Assignments	NOEs	Side Chain Accuracy (χ^1) (%)*	Side Chain Accuracy (χ^1+χ^2) (%)*
Supervised	HCNH	81	61
	HCNH+HCCH	81	67
TOCSY-NOESY	HCNH	75	60
	HCNH+HCCH	72	56
NOESY	HCNH	72	70
	HCNH+HCCH	70	56

*Side chain accuracies were computed for a fraction of correct dihedral placements (within 30° from the rotamer in the X-ray structure) in core side chains. The core side chains were selected in the X-ray structure using 10 Å^2 buried surface area threshold and further filtered by (i) rigid region (a-helix and b-sheet) in the secondary structure and (ii) the consistent side chain conformers in the crystallographic ensemble (PDB IDs 1P01, 1QRX,1SSX, 1TAL, 2ALP, 2H5C and 2ULL). Accuracy values were computed for 10 lowest-energy structures. The rotamer in the NMR ensemble is said to be accurate if it consistently lies within 30° from the same rotamer in X-ray structure. Buried surface area was calculated using PyMOL software (https://www.pymol.org).

Supplementary Table 6

Supplementary Table 6. NMR restraints and structural statistics for 10 lowest-energy RTT models.

**NMR distance and dihedral restraints**	
Distance restraints	
Total NOE	3447
Intra-residue	2024
Inter-residue	1423
Sequential ($\|i – j\|$ = 1)[**]	567
Medium-range ($\|i – j\|$ <= 4)[**]	612
Long-range ($\|i – j\|$ >= 5)[#]	244
Eliminated due to distance violations[%]	
less than 1 Å/structure	0.7 ± 0.64
between 1 Å and 2 Å/structure	0.0 ± 0.0
between 2 Å and 5 Å/structure	0.0 ± 0.0
above 5.0 Å/structure	0.0 ± 0.0
Total dihedral angle restraints*	248
ϕ	124
ψ	124
Ramachandran statistics[$]	
% of residues in favored regions	98.6
% of residues in allowed regions	100
outliers	0
**Structure statistics**[&]	
Average pairwise r.m.s. deviation (Å)	1.08
Average r.m.s. deviation to mean structure (Å)	0.71

[**]Distance restraints not used in structure calculations
[#]Distance restraints used in structure calculations by autoNOE-Rosetta
[%]Violations were calculated using 7 Å universal upper bound distance for unambiguous NOE restraints
[*]Residues that have good talos predictions
[$]Ramachandran statistics were calculated using MOLPROBITY over the lowest-energy models
[&]Computed over 10 lowest-energy structures for core residues 4-10, 15-27, 32-44, 50-70, 73-93, 96-112, 117-132

Supplementary Table 7

Supplementary Table 7. NMR restraints and structural statistics for 10 lowest-energy ms6282 models.

**NMR distance and dihedral restraints**	
Distance restraints	
Total NOE	2773
Intra-residue	1401
Inter-residue	1372
Sequential ($\|i - j\| = 1$)**	560
Medium-range ($\|i - j\| <= 4$)**	381
Long-range ($\|i - j\| >= 5$)#	431
Eliminated due to distance violations%	
less than 1 Å/structure	5.5 ± 0.67
between 1 Å and 2 Å/structure	0.0 ± 0.0
between 2 Å and 5 Å/structure	0.0 ± 0.0
above 5.0 Å/structure	0.0 ± 0.0
Total dihedral angle restraints*	226
ϕ	113
ψ	113
Ramachandran statistics$	
% of residues in favored regions	97.2
% of residues in allowed regions	100
outliers	0
**Structure statistics&**	
Average pairwise r.m.s. deviation (Å)	1.33
Average r.m.s. deviation to mean structure (Å)	0.9

**Distance restraints not used in structure calculations
#Distance restraints used in structure calculations by autoNOE-Rosetta
%Violations were calculated using 7 Å universal upper bound distance for unambiguous NOE restraints
*Residues that have good talos predictions
$Ramachandran statistics were calculated using MOLPROBITY over the lowest-energy models
&Computed over 10 lowest-energy structures for core residues 4-12, 16-24, 30-33, 42-45, 51-61, 64-75, 78-83, 88-96, 101-109, 125-144

Supplementary Table 8

Supplementary Table 8. NMR restraints and structural statistics for 10 lowest-energy aLP models.

**NMR distance and dihedral restraints**	
Distance restraints	
Total NOE	3234
Intra-residue	1625
Inter-residue	1609
Sequential ($\|i-j\| = 1$)[**]	708
Medium-range ($\|i-j\| <= 4$)[**]	361
Long-range ($\|i-j\| >= 5$)[#]	540
Eliminated due to distance violations[%]	
less than 1 Å/structure	7.5 ± 2.8
between 1 Å and 2 Å/structure	1.3 ± 1.35
between 2 Å and 5 Å/structure	0.1 ± 0.3
above 5.0 Å/structure	0.0 ± 0.0
Total dihedral angle restraints*	286
ϕ	143
ψ	143
Disulfide restraints[@]	3
Ramachandran statistics[$]	
% of residues in favored regions	94.8
% of residues in allowed regions	99.9
outliers	1
**Structure statistics**[&]	
Average pairwise r.m.s. deviation (Å)	0.94
Average r.m.s. deviation to mean structure (Å)	0.64

[**] Distance restraints not used in structure calculations
[#] Distance restraints used in structure calculations by autoNOE-Rosetta
[%] Violations were calculated using 7 Å universal upper bound distance for unambiguous NOE restraints
[*] Residues that have good talos predictions
[@] Disulfide restraints were used between the pairs of residues: 17 and 37; 101 and 111; 137 and 170
[$] Ramachandran statistics were calculated using MOLPROBITY over the lowest-energy models
[&] Computed over 10 lowest-energy structures for core residues 5-11, 14-25, 28-33, 35-37, 41-46, 49-58, 63-69, 78-81, 84-87, 97-103, 109-122, 127-134,144-149,152-160,179-191

Supplementary Table 9

Supplementary Table 9. NMR restraints and structural statistics for 10 lowest-energy nElt models.

**NMR distance and dihedral restraints**	
Distance restraints	
Total NOE	3454
Intra-residue	1896
Inter-residue	1558
Sequential ($\|i-j\| = 1$)**	746
Medium-range ($\|i-j\| <= 4$)**	434
Long-range ($\|i-j\| >= 5$)#	378
Eliminated due to distance violations%	
less than 1 Å/structure	1.7 ± 1.27
between 1 Å and 2 Å/structure	0.5 ± 0.92
between 2 Å and 5 Å/structure	0.4 ± 0.66
above 5.0 Å/structure	0.0 ± 0.0
Total dihedral angle restraints*	420
ϕ	210
ψ	210
Total RDC restraints	222
Ramachandran statistics$	97.6
% of residues in favored regions	99.9
% of residues in allowed regions	3
outliers	
**Structure statistics&**	
RDC Q-factors~	
R	0.25
Q	0.36
Average pairwise r.m.s. deviation (Å)	2.01
Average r.m.s. deviation to mean structure (Å)	1.36

**Distance restraints not used in structure calculations

#Distance restraints used in structure calculations by autoNOE-Rosetta

%Violations were calculated using 7 Å universal upper bound distance for unambiguous NOE restraints

*Residues that have good talos predictions

$Ramachandran statistics were calculated using MOLPROBITY over the lowest-energy models

&Computed over 10 lowest-energy structures for core residues 5-8, 11-18, 33-65, 67-81, 83-96, 100-117, 122-142, 157-160, 165-170, 179-181, 201-203, 208-211, 215-221, 225-229, 233-243

~RDC Q-factors were calculated over the core residues of the lowest-energy models

Supplementary Methods

4D-CHAINS

Installation

4D-CHAINS code and installation instructions are available at (https://github.com/tevang/4D-CHAINS)

Tutorial

To run 4D-CHAINS use the 4Dchains.py script. You can do all operations you wish with this script as long as you provide the appropriate protocol file, e.g.

4Dchains.py -protocol protocol.txt

You can find example files for nElt (248 residues) protein under the "**tutorials/nElt/**" folder. In that folder you will find the protocol file "**nElt_protocol.txt**", which contains all the input parameters for the program, and the following input files:

nElt.fasta is the sequence of the protein in fasta format

nElt_HSQC.list is the {N-H}-HSQC peak list in sparky format which consists of 3 columns:

<label> <N resonance> <HN resonance>

nElt_TOCSY.list is the 4D-TOCSY peak list in sparky format which consists of 5 columns:

<?-?-?-?> <H resonance> <C resonance> <N resonance> <HN resonance>

nElt_NOESY.list is the 4D-NOESY peak list in sparky format which consists of 6 columns (the last column can be omitted, but will decrease the accuracy of NOESY-based assignments):

<?-?-?-?> <H resonance> <C resonance> <N resonance> <HN resonance> <peak intensity>

nElt_TOCSY.list.curated this file is optional, it is the same as "nElt_TOCSY.list", but contains the supervised peak assignments for proofreading of 4D-CHAINS automatic assignments

nElt_NOESY.list.curated this file is optional, it is the same as "nElt_NOESY.list", but contains the supervised peak assignments for proofreading of 4D-CHAINS automatic assignments

The protocol file consists of compulsory and optional directives. The compulsory directives must be defined in order 4D-CHAINS to run, whereas the optional can be omitted. Briefly, the available protocol file "**nElt_protocol.txt**" will perform sequential and sidechain assignments of the protein using a 4D-TOCSY and a 4D-NOESY peak list. First, the N-H resonances are mapped to the protein sequence. These N-H resonances are then used to assign the aliphatic frequencies of the 4D-TOCSY peak list. Finally, the assignments from 4D-TOCSY are transferred to the respective 4D-NOESY peaks, and any missing aliphatic assignments are derived from the NOESY peak list. A short description of the most important directives is given below (for a detailed description of all available directives refer to the manual in the github repository).

fasta is the fasta sequence file.

HSQC is the {N-H}-HSQC peak list file.

4DTOCSY is the 4D-TOCSY peak list file.

4DNOESY is the 4D-NOESY peak list file.

user_4DTOCSY_assignedall is the optional 4D-TOCSY peaklist file with the supervised assignments. If this file, along with “user_4DNOESY_assignedall”, is provided, then 4D-CHAINS will proofread the automated TOCSY assignments and will add labels (<CORRECT>, <WRONG>).

user_4DNOESY_assignedall is the optional 4D-NOESY peak list file with the supervised assignments. If this file, along with “user_4DTOCSY_assignedall”, is provided, then 4D-CHAINS will proofread the automated NOESY assignments and will add labels (<CORRECT>, <WRONG>).

doNHmapping perform NH-mapping (“True”) or skip it (“False”).

doassign4DTOCSY perform assignment of 4D-TOCSY peak list (“True”) or skip it (“False”).

doassign4DNOESY perform assignment of 4D-NOESY peak list (“True”) or skip it (“False”).

user_4DTOCSY_assignedall is the optional 4D-TOCSY peaklist file with the supervised assignments. If this file, along with “user_4DNOESY_assignedall”, is provided, then 4D-CHAINS will proofread the automated TOCSY assignments and will add labels (<CORRECT>, <WRONG>).

user_4DNOESY_assignedall is the optional 4D-NOESY peak list file with the supervised assignments. If this file, along with “user_4DTOCSY_assignedall”, is provided, then 4D-CHAINS will proofread the automated NOESY assignments and will add labels (<CORRECT>, <WRONG>).

The following compulsory directives define the number of cycles for NH-mapping. All of them must have a consistent number of values separated by “,”. In each cycle, 4D-CHAINS does iterative NH-mapping using each time peptides of different length. It starts the first iteration by building long peptides, performs the NH-mapping, and then uses the mapped Amino Acid Index Groups (AAIGs) as restraints for the next iteration, which is conducted using shorter peptides. Each cycle consists of the number of iterations that is necessary to bring the peptide length form **first_length** to **last_length**. As such, the following values

first_length=6,6,6,6,6

last_length=4,4,4,4,3

instruct 4D-CHAINS to run 5 cycles, the first 4 cycles consist of 3 iterations, one with 6mer peptides, one with 5mers and one with 4mers, whereas the 5th cycle consists of 4 iterations, one with 6mers, one with 5mers, 4mers and finally one with 3mers.

In each cycle you can control the values of the following parameters:

mcutoff is a floating point number between 0.0-1.0 (percentage) that controls the occupancy rate of TOCSY-NOESY connectivities used in the buildup of chains.

zmcutoff is a floating point number (Z-score) that controls how many of the connected NOESY AAIGs satisfying the given mcutoff will be retained for chain formation.

zacutoff is a floating point number specifying the lower Z-score for an amino acid type prediction to be considered as valid.

To run the full protein assignment for the nEIt, type:

4Dchains.py -protocol nEIt_protocol.txt

At the end, you will find several output files and a directory named **4DCHAINS_workdir**, which contains all the intermediate files for backtracking (only for advanced users). The output files in the working directory are the following:

nEIt_HSQCnum.list is the original {N-H}-HSQC with the correct existing labels preserved and the other lines labeled as X1N-H, X2N-H, etc.

nEIt_TOCSYnum.list is the original 4D-TOCSY with N-H assignments or N-H labels

nEIt_NOESYnum.list is the original 4D-NOESY with N-H assignments or N-H labels

4DCHAINS_NHmap is the table with the N-H labels mapped to the protein sequence

4DCHAINS_assigned_NH_HSQC.sparky is the original {N-H}-HSQC peaklist with all the assignments made by 4D-CHAINS, in sparky format

4DTOCSY_assignedall.sparky is the original 4D-TOCSY peaklist with all the assignments made by 4D-CHAINS, in sparky format

4DTOCSY_assignedall.xeasy is the TOCSY-based assignments made by 4D-CHAINS, in xeasy format

4DNOESY_assignedall.sparky is the original 4D-NOESY with all the assignments made by 4D-CHAINS, in sparky format

4DNOESY_assignedall.proofread.xeasy is the TOCSY+NOESY-based or NOESY-based assignments made by 4D-CHAINS, in xeasy format. Since the files with supervised 4D-TOCSY and 4D-NOESY assignments were provided, this file contains at the end of each line comments like “<CORRECT>” or “<WRONG>”. If you hadn’t provided supervised assignments, this file would have been named “**4DNOESY_assignedall.xeasy”.**

From all the output files, the most important is “**4DNOESY_assignedall.proofread.xeasy”**, which will be passed as the input “chemical shift file” to autoNOE-Rosetta for protein structure prediction, as described below.

FLYA resonance assignment

Automated resonance assignments with FLYA were performed using the FLYA.cya script shown below.

FLYA.cya:

```
nproc=4
structurepeaks:=HCNH,HCCH
assignpeaks:=HCNH,HCCH,TOCSY
tolerance:=0.04,0.4,0.4,0.04
assigncs_accH:=0.04
assigncs_accC:=0.4
assigncs_accN:=0.4
shiftassign_population:=50
shiftassign_iterations:=15000
analyzeassign_group:=CONBB: N H CA C CB / CONSOLIDATED, CONALL: CONSOLIDATED, BB: N H CA C CB, ALL:*
randomseed  := 3771
command select_atoms
        atoms select "* - CZ ?H* @ARG - ?Z @LYS"
end
flya shiftreference=ref.prot structurepeaks=$structurepeaks assignpeaks=$assignpeaks
```

autoNOE-*Rosetta*

The following series of commands can be followed to set up and analyze Rosetta calculations exactly as was done for this benchmark. The user must first download both the CS-Rosetta Toolbox version 3.4 available under Downloads in http://csrosetta.chemistry.ucsc.edu and the Rosetta source-code under Rosetta license (https://www.rosettacommons.org). Instructions for compiling and/or installing CS-Rosetta toolbox can be found in http://csrosetta.chemistry.ucsc.edu and that of Rosetta can be found in https://www.rosettacommons.org.

Preparing files

Three initial files are required: a fasta file, chemical shift file (i.e. myShifts.xeasy), and NOE peak list(s). From these, the user may convert to the required formats using tools provided in the CS-Rosetta Toolbox.

.xeasy—>.prot

renumber_prot -s [#] -e [#] [myShifts.xeasy] > [myShifts.prot]

where -s indicates the first residue in the .xeasy, and -e indicates the last residue in the .xeasy

.prot—>.tab

prot2talos -fasta [myFasta.fasta] [myShifts.prot] [myShifts.tab]

The .prot will be used in the autoNOE protocol for NOE peak matching, while the .tab format is required for use with the TALOS-N program, included with the CS-Rosetta Toolbox, which is used to assess trimming of the termini, as well as fragment picking.

Trim flexible ends

talosn -in [myShifts.tab]

This output helps the user assess which residues are flexible and might consider trimming from the termini. Should the user decide to trim any residues, the steps above may be repeated to update the .prot and .tab files. The fasta should also be trimmed to reflect the adjusted sequence. The user may do this manually, or may create a new fasta using the following command:

talos2fasta [myShifts_trimmed.tab] > [myFasta_trimmed.fasta]

Convert peak lists to Rosetta format

Unassigned NOE peaks must be converted to Rosetta format using the following command:
clean_peak_file [hcNH.peaks] -skip [22] -cols [2 3 4 5 8] -names [h c N H I] -tol [0.02 0.20 0.20 0.02] >[hcNH.clean.peaks]

where –skip indicates how many lines come before the first NOE peak in the original file, -cols specifies which columns are relevant, and –names indicates the aforementioned columns. For instance, here we qualify columns 2, 3, 4, 5 as being chemical shifts pertaining to h, c, N, and H, and column 8 as the intensity of the peak. The flag –tol specifies the tolerance of each chemical shift column, respectively. Each peak list may be converted in this way, and multiple peak lists may be used with the autoNOE protocol.

Select 3-mer and 9-mer backbone fragments

Fragments are picked using the chemical shift information contained in the .tab file. Fragments used in this benchmark were picked using the optional flag –nohom, which excludes fragments derived from homologous structures. The output will contain a 3-mer and 9-mer fragment file, each ending in ".dat.gz".

pick_fragments -cs myShifts_trimmed.tab [-nohom]

Setting up directories and flag files

There are two commands used for setting up the directories and additional files needed for an autoNOE run: setup_target, and setup_autoNOE. The first command creates a directory with all the input files systematically organized, while the latter creates the run directory and automatically generates the flag files required for the Rosetta calculations and automatic assignment of NOE peaks.

setup_target -target [myTarget] -method autoNOE -frags [myTarget.frags*.dat.gz] -fasta [myFasta.fasta] -cs [myShifts_trimmed.tab] -disulfide_bonds [#1 #2 #3 #4 …] -peaks [hcNH.clean.peaks hcCH.clean.peaks] -shifts [myShifts_trimmed.prot] –rdc [myRDCs.rdc]

setup_autoNOE -target [myTarget]-method autoNOE -dir ~/autoNOE -job [myJobScript.production] -extras mpi –cst [05 10 25 50]

The default restraint weights for the "-cst" flag are 05, 10, 25, and 50, which this and previous benchmarks have found to be sufficient. The job script content will vary depending on the parallelization environment used. An example using the OpenGrid Sun Grid Engine parallel environment is as follows:

```
############################################################################
NSTRUCT=`echo $NSLOTS | awk '{print $1-3}'`
ROSETTA3=$/home/user/Rosetta
PATH=$PATH:$ROSETTA3/main/source/bin
LOGS=logs_`echo $PBS_JOBID | awk -v FS="." '{print $1}'`
mkdir -p $LOGS

echo ""
echo $PATH
echo $LD_LIBRARY_PATH
echo $TMPDIR
echo ""
echo "Executing on : $HOSTNAME"
echo "Number of hosts operating on : $NHOSTS"
echo "Number of queued slots in use for parallel job: $NSLOTS"
# where $NSLOTS is as submitted to -pe command to the OpenGrid SGE scheduler
echo ""
echo "Running on $NSLOTS cpus …"
#
#Current Rosetta binaries
EXE=/home/user/Rosetta/main/source/bin/minirosetta.mpi.linuxgccrelease
CMDLINE="-out:level 300 -mute all_high_mpi_rank_filebuf -out:mpi_tracer_to_file $LOGS/log -database
/home/userRosetta/main/database -out:file:silent decoys.out @flags_denovo @flags_autoNOE
@flags_iterative -run:archive"
CYCLES="-out:nstruct $NSTRUCT "
echo $EXE
echo $CMDLINE
echo $CYCLES

mpiexec -n $NSLOTS $EXE $CMDLINE $CYCLES

exit
############################################################################
```

Initializing NOE assignments

Before the autoNOE protocol is started, initial NOE assignments are created using the command "source initialize_assignments_phaseI.sh" from within the run sub-directory of each restraint-weight directory. This will create four peak assignment files: noe_auto_assign.cst.centroid, noe_auto_assign.cst.filter.centroid, noe_auto_assign.cst, and noe_auto_assign.cst.filter. To initialize the assignments of restraint-weight directories, the following commands may be used:

```
cd cst_XX/run
source initialize_assignments_phaseI.sh
```

Start run

Calculations are started from within each run sub-directory. It is recommended to run calculations using at least the four default restraint weights to assess which works best for a given target. For instance, depending on the quality or confidence of initial input, one may see better results with either harsh or more lenient weights. For this benchmark, we tested all default weights (plus weight 100 for nElt), and found that higher restraint weights worked best given the high quality of the automatically assigned chemical shifts, namely weights 25, 50 and 100. The command used to begin the Rosetta calculations depends on the parallelization environment used. An example using the OpenGrid Sun Grid Engine parallel environment is as follows:

```
qsub –pe orte 100 myJobScript.production
```

where 100 is the number of cores to be used in parallel for this run.

Processing and analyzing a run

Once all runs for a given target have completed (i.e. all restraint weight run have completed), a quick check for the best performing run can be performed using the following command from within autoNOE/myTarget/:

```
autoNOE_select_final_run
```

Further documentation of autoNOE_select_final_run may be found at http://csrosetta.chemistry.ucsc.edu.

A more detailed analysis can be performed using a variety of tools offered with the CS-Rosetta Toolbox.

Select the 10 lowest scoring structures

Below is an example of rescoring decoys from within the fullatom_pool sub-directory:

```
score_jd2.macosclangrelease -in:file:silent decoys.out -evaluation:rmsd_target myKnownStructure.pdb -out:file:silent rescored_decoys.out
```

To extract the lowest ten scoring decoys based on the total Rosetta energy scores:

```
extract_decoys rescored_decoys.out -formula 'score-atom_pair_constraint-rdc' -N 10 -verbose 0 > rescored_low_10.out
```

To pack ten lowest-energy structures into a PDB bundle:

```
pack_pdbs -silent rescored_low_10.out > final.pdb
```

To extract PDBs of the ten lowest-energy structures:

```
extract_pdbs.macosclangrelease -in:file:silent rescored_low_10.out
```

To extract PDBs from the PDB bundle:

```
cat final.pdb | unpack_pdbs -remark ROSETTA-TAG
```

To print the total score of the ten lowest-energy structures, sorted by total Rosetta energy (field numbers may vary):

```
cat rescored_low_10.out |grep SCORE |awk '{print $2}' |sort
```

Ensemble convergence statistics

```
ensemble_analysis.macosclangrelease -chemical:patch_selectors replonly -in:file:silent rescored_low_10.out -wRMSD 2 -calc:rmsd
```

NOE assignment statistics (from within the run directory)

Final static NOE assignments and statistics can be generated with the two following commands:

```
source final_assignments.sh ../fullatom_pool/rescored_low_10.out
```

```
noeout2txt -peaks final_assignment/NOE_final.out -split_level 0
```

Converting to NEF for data deposition

After the structure calculations are complete and are ready to be deposited to a database, NMR restraint data that was used for structure calculation can be converted to NEF format and uploaded to the database using following commands:

nef -fasta [myFasta.fasta] -cs [myShifts_trimmed.tab] -prot [myShifts_trimmed.prot] -restraint [myRestraints.cst] -rdc [myRDC.rdc] -peaks [hcCH.clean.peaks hcNH.clean.peaks] -NEF myNefFile.nef

Extracting data from NEF

Any compliant NEF format file that contains all the appropriate sections can be used to extract respective file and trigger the structure calculations using CS-Rosetta.

Extracting fasta: The following command is used to extract sequence information from the NEF file and write it to myFasta.fasta file.

nef2fasta –NEF [myNefFile.nef] –fasta [myFasta.fasta]

Extracting shifts: One of the commands below can be used to extract chemical shifts from NEF file. To extract shifts into prot file or tab file, we can use nef2prot or nef2talos commands to write chemical shift data into myShifts.prot or myShifts.tab file respectively.

nef2prot –NEF [myNefFile.nef] –prot [myShifts.prot]

nef2talos –NEF [myNefFile.nef] –tab [myShifts.tab]

Extracting RDCs: We can extract RDC data if available from NEF into myRDC.rdc using nef2rdc as given below.

nef2rdc –NEF [myNefFile.nef] –rdc [myRDC.rdc]

Extracting peaks: nef2peaks command is used to get the peaks from the NEF and write them into corresponding output files.

nef2peaks –NEF [myNefFile.nef] –peaks [myPeaks_hcNH.peaks myPeaks_hcCH.peaks]

Extracting restraints (or csts): We can extract the assigned NOE restraints into the cst file using nef2restraint command as given below:

nef2restraint – NEF [myNefFile.nef] –restraint [myRestraints.cst]

CYANA structure calculations

Automated NOE assignment and structure calculation with CYANA were performed using the AUTO.cya script shown below.

AUTO.cya:

```
nproc=4
peaks       := HCNH,HCCH    # names of peak lists
prot        := aLPmanual.prot   # names of chemical shift lists
constraints := talosn.aco,ssbond.upl,ssbond.lol    # additional (non-NOE) constraints
tolerance   := 0.04,0.4,0.4,0.04    # chemical shift tolerances
calibration_dref := 4.2,4.2  # NOE calibration parameters
upl_values  := 2.4,6.0
structures  := 100,20                        # number of initial, final structures
steps       := 20000                         # number of torsion angle dynamics steps
seed        := 210005                   # random number generator seed
noeassign peaks=$peaks prot=$prot autoaco
```
